# Molecular Engineering of Recombinant Protein Hydrogels: Programmable Design and Biomedical Applications

**DOI:** 10.3390/gels11080579

**Published:** 2025-07-26

**Authors:** He Zhang, Jiangning Wang, Jiaona Wei, Xueqi Fu, Junfeng Ma, Jing Chen

**Affiliations:** 1 National Engineering Laboratory for AIDS Vaccine, School of Life Sciences, Jilin University, Changchun 130012, China; zhanghe8221@mails.jlu.edu.cn (H.Z.); fxq@jlu.edu.cn (X.F.); 2 College of Plant Science, Jilin University, Changchun 130062, China; wangjn8221@mails.jlu.edu.cn; 3 Faculty of Animal Science and Technology, Jilin Agricultural University, Changchun 130118, China; naia1212@163.com

**Keywords:** recombinant protein, hydrogel, molecular engineering, programmable biomaterials, physical and chemical crosslinking, biomedical applications

## Abstract

Recombinant protein hydrogels have emerged as transformative biomaterials that overcome the bioinertness and unpredictable degradation of traditional synthetic systems by leveraging genetically engineered backbones, such as elastin-like polypeptides, SF, and resilin-like polypeptides, to replicate extracellular matrix (ECM) dynamics and enable programmable functionality. Constructed through a hierarchical crosslinking strategy, these hydrogels integrate reversible physical interactions with covalent crosslinking approaches, collectively endowing the system with mechanical strength, environmental responsiveness, and controlled degradation behavior. Critically, molecular engineering strategies serve as the cornerstone for functional precision: domain-directed self-assembly exploits coiled-coil or β-sheet motifs to orchestrate hierarchical organization, while modular fusion of bioactive motifs through genetic encoding or site-specific conjugation enables dynamic control over cellular interactions and therapeutic release. Such engineered designs underpin advanced applications, including immunomodulatory scaffolds for diabetic wound regeneration, tumor-microenvironment-responsive drug depots, and shear-thinning bioinks for vascularized bioprinting, by synergizing material properties with biological cues. By uniting synthetic biology with materials science, recombinant hydrogels deliver unprecedented flexibility in tuning physical and biological properties. This review synthesizes emerging crosslinking paradigms and molecular strategies, offering a framework for engineering next-generation, adaptive biomaterials poised to address complex challenges in regenerative medicine and beyond.

## 1. Introduction

In recent years, biomedical engineering and materials science have converged to catalyze transformative advances in intelligent biomaterials. Among these, hydrogels stand out for their biomimetic properties and programmable functions, making them indispensable platforms in both regenerative medicine and precision therapeutics [1,2,3,4]. Traditional synthetic hydrogels, such as polyacrylamide (PAAm) and polyethylene glycol (PEG) systems, have demonstrated impressive mechanical tunability [5,6]. However, their inherent bioinertness, unpredictable degradation profiles, and limited biological functionality significantly hinder their utility in complex and dynamic physiological settings. To address these challenges, protein-based hydrogels are designed to emulate critical aspects of the native extracellular matrix (ECM), integrating enzyme-responsive domains and cell-adhesive ligands within a highly hydrated three-dimensional (3D) scaffold [7,8,9,10]. These systems employ a hierarchical crosslinking architecture, initially stabilized by reversible interactions such as hydrogen bonds and hydrophobic forces, and subsequently reinforced by dynamic covalent bonds (e.g., Schiff base reactions, Michael-type additions, or light-activated linkages), to simultaneously achieve mechanical integrity and ECM-mimetic biofunctionality [11,12,13,14,15,16,17].

Natural protein hydrogels, including those derived from collagen, fibrin, gelatin, and SF, have long been employed in biomedical applications due to their inherent biocompatibility and biodegradability. However, these materials often exhibit uncontrollable immunogenicity, low programmability, and batch heterogeneity. In addition, extraction and purification processes for natural proteins are time-consuming and costly, posing significant challenges for large-scale manufacturing and standardization. As a versatile alternative, recombinant protein hydrogels have emerged and dramatically expanded due to recent breakthroughs in synthetic biology and protein engineering [18]. By leveraging genetic engineering to precisely tailor molecular architectures, recombinant protein hydrogels achieve superior spatiotemporal control of bioactivity, predictable degradation profiles, and integrated multifunctionality—outperforming traditional synthetic hydrogel systems.

**Figure 1:**
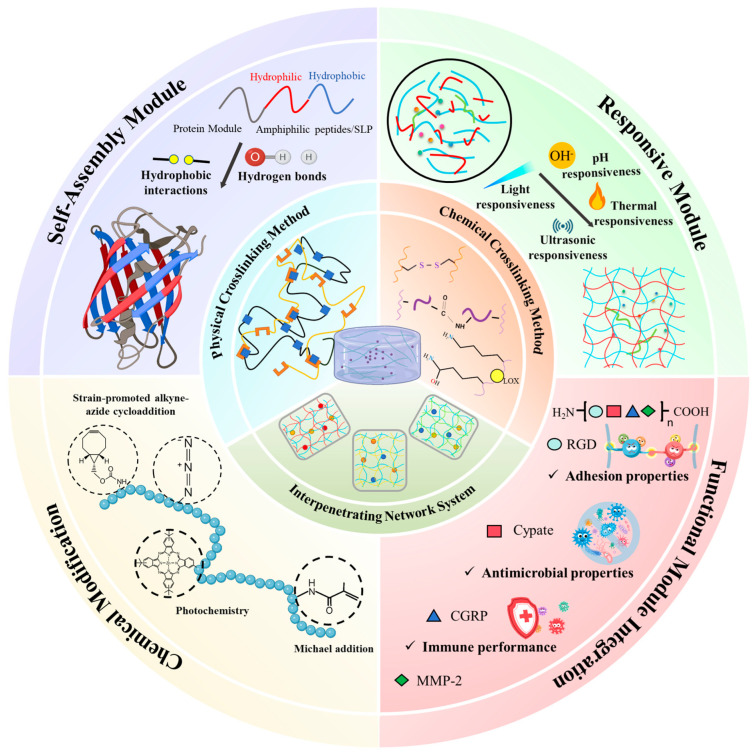
Molecular engineering strategies for constructing recombinant protein hydrogels with tailored structure and function. The central panel depicts key crosslinking mechanisms, including physical and chemical methods, as well as interpenetrating network systems that synergistically enhance mechanical properties and dynamic behavior. Surrounding quadrants represent four molecular design approaches: self-assembly driven by sequence-encoded hydrophobic interactions and hydrogen bonding, stimuli-responsiveness tuned through environmental triggers such as pH, light, temperature, and ultrasound, chemical modifications using click-chemistry and photochemistry reactions, and functional motif integration to endow the hydrogel with bioactivities including adhesion, antimicrobial function, and immune modulation. These strategies collectively enable precise control over the hierarchical organization, responsiveness, and biofunctionality of recombinant protein hydrogels.

This review synthesizes recent advances in recombinant protein hydrogel research, emphasizing dynamic network construction and functional integration (Figure 1). It systematically covers crosslinking mechanism design principles, modular sequence engineering strategies, and emerging interdisciplinary applications, with particular emphasis on domain-guided self-assembly optimization and functionalization for bioactivity control. Mechanistic insights into the synergistic interplay between non-covalent interactions (hydrogen bonding, hydrophobic associations) and enzyme-responsive covalent networks are provided to illustrate how modular protein sequences can be programmed for precise mechanical and degradation profiles. Innovative applications, including spatiotemporal therapeutic delivery for vascularized wound healing, microenvironment-responsive drug release systems, and mechanochemically integrated bioprinting scaffolds, are examined to demonstrate the breadth of functional integration. Looking ahead, artificial intelligence-assisted modular design, multi-omics-driven functional convergence, and streamlined clinical translation pathways will drive the next phase of development. Together, these advances foreshadow a transformation of recombinant protein hydrogels from customizable scaffolds into truly intelligent bionic platforms ready for clinical translation.

## 2. Key Protein Backbones in Recombinant Hydrogel Systems

Current research has revealed that many natural proteins contain tandem repetitive motifs, i.e., arrays of similar amino acid sequences, that confer unique mechanical properties and often act as spacer elements between discrete functional domains. Leveraging these motifs, macromolecular bioengineering has created diverse protein-based hydrogel platforms, especially from elastin-like polypeptides (ELPs), silk fibroin (SF), resilin-like polypeptides (RLPs), and mussel foot proteins (MFPs) [19,20,21,22,23,24,25]. By precisely editing amino acid sequences, tuning intermolecular interactions, and incorporating environmental responsiveness, these engineered proteins yield hydrogels with adjustable mechanics, reversible phase behavior, and robust multi-interface adhesion.

### 2.1. ELPs

Elastin is a key ECM protein in vertebrate connective tissues, notable for its exceptional elasticity and resilience [23]. As an intrinsically disordered protein (IDP), tropoelastin monomers lack a fixed tertiary structure until assembly, enabling them to function as molecular springs that impart tissues with superior deformation recovery and efficient energy storage–release cycles [26]. In vivo, lysyl oxidase (LOX) catalyzes the oxidative deamination of specific lysine residues in tropoelastin, generating aldehydes (allysine) that spontaneously condense into tetrafunctional desmosine and isodesmosine crosslinks, as well as bifunctional allysine-aldol bonds, to stabilize the mature elastin fiber network [27]. This extensive crosslinking produces an insoluble, durable elastin matrix, responsible for the remarkable elasticity and longevity of elastic fibers in tissues such as the skin, lung, and blood vessels.

Inspired by these native features, researchers have engineered ELPs as functional scaffolds for recombinant protein hydrogels. ELPs consist of tandem repeats of the pentapeptide motif “(VPGXG)_n_”, where “X” can be any amino acid except proline and “n” denotes the number of repeats [28]. By varying the identity of the guest residue “X” and the repeat length “n”, it is possible to finely tune the lower critical solution temperature (LCST) and mechanical properties of ELP hydrogels [29]. The thermoresponsive behavior of ELPs adheres to thermodynamically reversible phase transition principles: below the transition temperature (Tt), ELP chains are highly solvated via hydration; above Tt, hydrophobic interactions drive cooperative phase separation, resulting in reversible coacervation and self-assembly into dense, elastic networks [30].

Thermodynamic parameters governing the ELP phase transition include intramolecular hydrogen bonding, side-chain hydrophobicity, and conformational entropy, all of which can be modulated by the choice of guest residue and repeat number. For example, substituting more hydrophobic residues in the “X” position lowers Tt by reducing solvation energy, whereas increasing the repeat count “n” enhances intermolecular interaction strength and stiffness. These dual design levers grant precise, molecular-level control over ELP hydrogel behavior, enabling customization for specific biomedical applications [31,32]. Experimental studies have demonstrated programmable tuning of ELP hydrogel properties by engineering chain length and amino acid sequences [33]. Advanced computational approaches, including high-throughput molecular dynamics simulations, have been employed to elucidate the influence of peptide sequence, chain length, and ionic strength on ELP conformational ensembles and coacervation behavior [34,35,36]. These in silico frameworks facilitate virtual screening of ELP libraries, linking sequence variations to macroscopic properties and thus accelerating the design of intelligent ELP-based hydrogels. The virtual screening platform both deciphers how ELP sequence features govern multiscale structure–function relationships and generates sequence-programmable ELP libraries, thereby providing an integrated innovation toolkit that bridges molecular design and performance prediction for next-generation intelligent protein hydrogels.

### 2.2. RLPs

Resilin, a critical elastomeric protein in insect exoskeletal systems, has become a pivotal focus in biomimetic material research owing to its exceptional superelasticity and energy storage properties [21,37]. Structural elucidation of natural resilins, such as Dros16 from Drosophila melanogaster (core sequence: (GGRPSDSYGAPGGGN)_n_) and An16 from Anopheles gambiae (core sequence: (AQTPSSQYGAP)_n_), has facilitated the development of recombinant RLPs that replicate native mechanical functionalities [38,39,40]. Notably, sequence heterogeneity inherited from insect-derived resilin templates introduces species-specific divergence in thermoresponsive properties, necessitating precise functionalization strategies governed by sequence–performance relationships [41]. Advanced sequence engineering enables programmable design of RLPs with tunable mono-/biphasic Tt, where phase transitions may manifest as thermodynamically reversible phenomena or stabilize via irreversible aggregation pathways [42].

Notably, investigations demonstrate temperature-dependent conformational dynamics in RLPs: Elevated temperatures trigger hydrophobicity-driven molecular aggregation, accompanied by characteristic transitions in secondary structures from disordered states to β-sheet-rich configurations. Specific RLP systems exhibit upper critical solution temperature (UCST) behavior, undergoing reversible phase separation below defined thermal thresholds [43]. At the mechanistic level, RLPs and ELPs exhibit conserved concentration-dependent phase transition behavior, with Tt inversely proportional to concentration [44]. However, their salt-responsive characteristics diverge fundamentally: in RLPs, low-ionic-strength conditions (<0.1 M NaCl) enhance intermolecular interactions through charge shielding effects, elevating Tt. At 0.1–1 M NaCl, Tt increases by 10–14 °C in aqueous systems and approximately 5 °C in phosphate-buffered saline (PBS), demonstrating a distinctive “salting-in” effect that contrasts with the “salting-out” behavior of ELPs under analogous conditions [44,45]. Mechanistic analyses reveal strict adherence of RLP salt effects to the Hofmeister anion series, where anions regulate solubility via specific interactions with charged peptide moieties. Crucially, anion charge density and polarizability emerge as dominant parameters governing interaction intensity [46].

Recombinant synthesis of RLPs (as well as ELPs) is commonly achieved through recursive directional ligation, a method that enables precise control over gene sequence and length by tandemly assembling short gene segments [47]. These constructs are typically expressed in *Escherichia coli* (*E. coli*), where optimized fermentation strategies (e.g., lactose-induced expression) have achieved yields up to 300 mg/L [48]. Alternatively, the *Brevibacillus choshinensis* secretion system offers high-yield, column-free purification, producing up to 530 mg/L of RLPs [49]. In essence, RLPs, characterized by their distinctive thermal- and salt-responsive behaviors, offer programmable structures and tunable functionalities. These attributes make them highly promising candidates for various biomedical applications, including tissue engineering, drug delivery, and the development of responsive hydrogels.

### 2.3. Recombinant SF

SF, primarily sourced from the domesticated silkworms (*Bombyx mori*) and orb-weaving spider, has garnered significant attention in biomaterial research due to its exceptional mechanical properties, including high tensile strength, remarkable toughness, and low density [50]. The underlying molecular architecture of SF consists of alternating crystalline and amorphous blocks: β-sheet-forming sequences such as (Ala)_n_ and (Gly-Ala)_n_ impart rigidity and load-bearing capacity through extensive intermolecular hydrogen bonds. In contrast, the amorphous regions, which are enriched in motifs like (Gly-Pro-Gly-X-X)_n_ or (Gly-Gly-X)_n_, where “X” is frequently glutamine, afford chain mobility and extensibility, enabling molecular slippage and energy dissipation under strain. This hierarchical combination of stiff β-sheets interspersed with flexible linkers yields a synergistic balance of strength and toughness [51].

Despite spider dragline silk’s superior tensile properties, it remains difficult to produce at scale due to challenges in recombinant expression and post-translational processing [50]. Conversely, SF from *Bombyx mori* can be readily extracted from cocoons, but even this natural source suffers from batch-to-batch variability and limited tunability of its physicochemical properties, motivating the development of recombinant silk protein. Through genetic engineering, researchers can precisely control the length of β-sheet-forming repeats, the ratio of crystalline to amorphous segments, and the introduction of functional domains (e.g., RGD for cell adhesion), thereby programming silk proteins for desired material properties.

The self-assembly and secondary structure adoption of recombinant silk proteins are governed by a hierarchy of factors: core repetitive sequences provide the β-strand propensity; non-repetitive terminal domains (NTDs/CTD) facilitate nucleation and fiber formation via specific molecular recognition motifs; and environmental parameters (pH, ionic strength, shear stress) dynamically regulate the kinetics and morphology of assembly [52,53,54]. By leveraging this mechanistic understanding, recombinant silk proteins have been fabricated into a multitude of formats, including fibers, films, porous 3D scaffolds, and microcapsules. Fibrous constructs often achieve Young’s moduli ranging from 0.1 GPa to 20 GPa (depending on post-spin stretching and crystallization), while 3D porous scaffolds—tuned via salt leaching or freeze-drying—exhibit adjustable compressive moduli spanning 0.1–100 kPa, matching mechanical requirements of soft tissues such as cartilage and muscle [55,56].

### 2.4. MFPs

Marine mussels secrete several key adhesive proteins, i.e., MFPs from their foot glands, to form a functionally stratified architecture within their byssal threads [19]. These proteins are characterized by a high content of 3,4-dihydroxy-L-phenylalanine (DOPA), a catecholic amino acid with a bisphenol structure, which underpins mussels’ multivalent interfacial adhesion through oxidative covalent crosslinking, dynamic covalent bonds, hydrogen bonding, and metal-coordination interactions [57]. Notably, the MFP system integrates synergistic functional motifs: dense cationic lysine residues and anionic phosphoserine groups form short-range cation–π interaction networks. This dynamic crosslinking mechanism, mediated by aromatic rings, charged moieties, and environmental ions, enhances the structural integrity of byssal threads and provides hierarchical molecular strategies for achieving robust adhesion and mechanical resilience in complex intertidal environments [58].

Recombinant production of MFPs has been explored to overcome limitations in natural extraction, with various expression systems such as *E. coli* and Pichia pastoris employed to produce functional MFPs. Challenges in recombinant expression include achieving proper post-translational modifications, particularly the hydroxylation of tyrosine residues to DOPA, which is critical for adhesive functionality. Coexpression with tyrosinase enzymes has been utilized to enhance DOPA content in recombinant MFPs, improving their adhesive properties [59]. To enhance expression yields and functional properties, hybrid proteins combining sequences from different MFPs have been developed. For example, a fusion protein comprising MFP-5 at each terminus of MFP-3, known as fp-535, demonstrated improved adhesion strength and higher yield in bioreactor cultures [60].

Inspired by the physicochemical versatility of mussel proteins, engineering MFP-based hydrogels yields exceptional functionalities, including superior adhesive strength, photothermal responsiveness, biocompatibility, injectability, stretchability, and self-healing properties [61]. For instance, Luo et al. demonstrated that tyrosinase-catalyzed hydrogels derived from MFP-3 exhibit dual functionality: these materials achieve stable underwater adhesion on hydrated biological tissues while enabling stem cell encapsulation and delivery. The mechanical stiffness of the hydrogels can be precisely modulated by polymer concentration. Experimental validation confirmed their remarkable underwater adhesion to porcine skin tissue and excellent cytocompatibility in 3D stem cell culture systems, establishing a novel platform for dynamic biointerface engineering [62]. These advancements underscore the potential of recombinant MFPs as versatile biomaterials in biomedical applications, including tissue engineering, wound healing, and the development of bioadhesive coatings.

### 2.5. Other Engineered Proteins

Recent advances in biomaterials prepared by other recombinant proteins, such as keratin, gelatin, fibrin, and collagen-like peptides (CLPs), have also highlighted their unique properties for biomedical applications. Keratin, distinguished by its exceptional mechanical strength, thermal stability, and inherent biological functions (e.g., hemostasis promotion and wound-healing facilitation), has garnered significant attention in biomaterial research. The superiority of recombinant keratin stems from its molecular architecture enriched with cysteine residues, which enable dynamic disulfide bond networks to confer self-healing capability and tunable mechanical responsiveness [22]. Chen et al. successfully engineered type I (K35, K36) and type II (K81, K85) recombinant keratins via genetic modification, utilizing indomethacin as a model drug to validate the controlled-release performance of the hydrogel system. Experimental findings revealed that these keratin hydrogels exhibited excellent mechanical strength and high elasticity, demonstrating significant potential for applications in drug delivery and tissue engineering [63].

Recombinant gelatin features a molecular scaffold composed of characteristic (Gly-X-Y)_n_ triplet repeats, a biomimetic architecture that closely mimics the amino acid arrangement of native collagen, endowing the material with ECM-mimetic topological characteristics [24]. In vitro co-culture experiments with adipose-derived stem cell (ASC)- and recombinant collagen peptide (RCPhC1)-integrated hydrogels revealed exceptional biocompatibility, with cell viability sustained above 85% throughout the culture period. Microscopic analysis further demonstrated that ASCs within the hydrogels exhibited pronounced morphological extension [64]. Beyond these recombinant systems, fibrin-based and CLP-derived hydrogel platforms have also attracted substantial interest in biomedicine due to their high biomimetic fidelity [25,65].

These diverse protein architectures not only provide a rich repository of functional motifs but also offer versatile platforms for designing hydrogels with tailored properties. The thermoresponsive phase transitions of ELPs, hierarchical assembly of SF, salt-responsive behavior of resilin, multimechanistic adhesion of mussel proteins, and biofunctional integration of other recombinant proteins collectively establish a molecular toolkit for intelligent biomaterials. Contemporary protein-based hydrogel research has transcended conventional material limitations through the synergistic integration of gene editing, computational modeling, and multiscale fabrication technologies, enabling precise mapping from molecular sequences to macroscopic performance.

## 3. Classification and Principles of Crosslinking Mechanisms in Recombinant Protein Hydrogels

The crosslinking mechanism serves as the central determinant governing the mechanical properties, dynamic responsiveness, and biological functionalities of hydrogels [66,67,68,69,70,71,72,73,74]. To fully harness the potential of recombinant proteins in hydrogel applications, a comprehensive understanding of their crosslinking mechanisms is essential. According to the type of molecular force and the mode of crosslinking, these mechanisms can be broadly categorized into two types: physical and chemical crosslinking.

### 3.1. Physical Crosslinking Method

Physically crosslinked recombinant protein hydrogels form a dynamically tunable biomaterial network through non-covalent mechanisms, including electrostatic interaction, hydrogen bonding, van der Waals forces, and hydrophobic interaction [75,76].

Electrostatic interactions: These interactions confer reversible crosslinking behavior and programmable charge dynamics to hydrogels by modulating the density and spatial distribution of charged groups (e.g., carboxylate, amino residues). For instance, the recombinant silk protein system achieves spatiotemporally controlled release of the anticancer drug doxorubicin through dynamic charge regulation [77,78].

Hydrogen bonds: The dynamic reversibility of hydrogen bonds plays a central role in molecular bridging and mechanical adaptation. For example, SF forms high-density crosslinking through the hydrogen bond network in the random coil region, combined with the mechanical support of the β-sheet crystalline region, achieving a compression modulus that matches natural tissue. Additionally, the chitosan–SF composite system synergistically regulates swelling behavior and pro-angiogenic activity through hydrogen bonds, revealing potential in vascularized tissue regeneration [79,80].

Van der Waals forces: Although individually weak, van der Waals forces are indispensable in multi-level structure assembly and self-healing function [75]. For example, the β-sheet of silk protein maintains the spacing of nanocrystalline regions through van der Waals force, supporting mechanical stability. Self-healing hydrogels designed based on this mechanism can adapt to the periodic stress of joint motion, reducing the risk of material failure due to fatigue [81].

Hydrophobic interactions: These interactions drive the directional aggregation of hydrophobic groups (such as alanine, valine) through entropy, dominating the folding and self-assembly processes of proteins. The gelation of SF relies on hydrophobic interaction to form a β-sheet conformation. Furthermore, SF/polylactic acid (PLA)-PEG-PLA composite systems achieve sustained release of the anti-inflammatory drug indomethacin through regulation of hydrophobic interaction, highlighting advantages in drug delivery [82,83].

The synergistic effects of these non-covalent mechanisms provide multi-level functional regulation for recombinant protein hydrogels. Based on the various non-bonding forces described above, physical crosslinking methods of recombinant protein hydrogels can be divided as outlined below.

#### 3.1.1. One-Component Coiled-Coil Self-Assembly

Coiled-coil motifs are fundamental protein self-assembly structures formed by the superhelical winding of two or more α-helices into stable configurations. Their defining feature is the periodic arrangement of heptad repeat sequences (abcdefg)_n_, where hydrophobic residues, typically leucine, in the a and d positions drive helix assembly through hydrophobic interactions. This hydrophobic-driven assembly underpins the structural basis of the leucine zipper domain [84]. In constructing supramolecular networks, the directional assembly characteristics of coiled coils provide a molecular design basis for regulating the stiffness, viscoelasticity, and degradation behavior of hydrogels.

Engineered protein hydrogels based on leucine zippers often involve a triblock structure: a central water-soluble polyelectrolyte domain (C10) flanked by self-assembly domains (A) at both ends, forming a modular configuration of AC10A [85]. Such physically crosslinked systems can form dynamic networks in response to environmental conditions like pH and temperature. However, they are limited by structural defects such as reversible crosslinking characteristics and intramolecular cyclization, often exhibiting issues like insufficient mechanical strength and rapid degradation [86]. To address these challenges, researchers have employed multidimensional optimization strategies: enhancing crosslinking stability by precisely tuning the association kinetics of coiled-coil motifs; redesigning the chemical composition of the intermediate connection domain to regulate the network topology; and optimizing the overall molecular weight distribution to balance material strength and dynamic responsiveness [87,88]. For instance, replacing the self-assembled end block in AC10A with a new coiled-coil sequence (P) effectively inhibits the intramolecular cyclization, significantly improves the mechanical stability of the hydrogels, and slows the degradation rate [89]. These advancements underscore the potential of coiled-coil-based hydrogels in biomedical applications, offering customizable platforms for tissue engineering, drug delivery, and regenerative medicine.

#### 3.1.2. Stimuli-Responsiveness

Recombinant protein hydrogels exhibit intelligent responsiveness to external stimuli, primarily mediated by reversible non-covalent interactions. These dynamic physical crosslinking networks confer sensitivity to temperature, pH, and magnetic fields, enabling precise modulation of sol–gel transitions, mechanical properties, and drug release kinetics in response to environmental changes.

Temperature-responsive recombinant protein hydrogels achieve tunable crosslinking through controllable covalent or reversible interactions, enabling precise regulation of crosslinking density and mechanical properties at specific temperature thresholds. Temperature modulation governs the transition between reversible and irreversible crosslinking modes, enhancing design flexibility for dynamic tissue engineering scaffolds and controlled drug delivery systems. For instance, Duan et al. engineered thermoresponsive hydrogels by fusing an ELP sequence (VPGVG)_n_ with a chimeric elastin module (GR)_4_, composed of globular protein GB1 (G) and random-coil-sequence resilin (R) domains. The resulting (VPGVG)_n_-(GR)_4_ self-assembled into 3D hydrogels, where ELP side chains underwent temperature-induced phase transitions, thereby enhancing mechanical properties and enabling dynamic tunability of the hydrogel network [33]. Furthermore, Youn et al. developed a novel thermoreversible injectable hydrogel system (P-S hydrogels) through the physical blending of Pluronic F-127 (PF) and SF. This system remains in an injectable sol state at 4 °C, and undergoes gelation within 10 min at a physiological temperature of 37 °C, showing excellent temperature-sensitive response characteristics.

pH-responsive recombinant protein hydrogels exploit changes in physiological or local microenvironmental pH to reversibly regulate crosslinking states, thereby imparting dynamic adaptability and precise targeting capabilities. At specific pH thresholds, intermolecular electrostatic interactions or enhanced hydrogen bonding networks are triggered, inducing protein conformational changes that lead to the formation of physical crosslinking networks. For example, Zhou’s team developed Col-APG-Cys@HHD nanocomposite hydrogels. Through innovative molecular interface engineering and pathological microenvironment-responsive design, collagen and recombinant human albumin nanoparticles (HHD NPs) were utilized as the core components. A 3D network was constructed via dynamic covalent crosslinking of aldehyde-functionalized PEG (APG6K), forming a bifunctional interface with both adhesive and antiadhesive properties. The hydrogel exhibited controlled drug release behavior across a pH gradient (6.5 to 5.5), facilitating its dissociation in acidic tumor environments, subsequent release into tumor cells, and effective drug delivery [90].

Magnetic field-responsive recombinant protein hydrogels offer advantages such as deep tissue penetration and excellent biosafety, providing a promising platform for non-invasive and precise drug delivery. Notably, magnetic fields can penetrate deep into living tissues and are well tolerated by the human body compared to other stimuli such as temperature and electric fields [91,92,93]. By integrating biocatalysis and molecular self-assembly techniques, Conte et al. developed a novel paradigm for the controllable self-assembly of magnetic nanoparticles (MNPs) mediated by thermophilic protease catalysis. This system drives the amide bond condensation reaction between Fmoc-T and F-NH2 through enzymatic oxidation, successfully synthesizing the Fmoc-TF-NH2 gelator, which subsequently self-assembles into a hydrogel. This process triggers the radial growth of β-sheet nanofibers around MNPs, forming a unique “hub-and-spoke” supramolecular hydrogel. This method significantly enhances the shear strength and stability of the hydrogel system, with its formation and overall structure being externally controllable via applied magnetic fields [94].

The performance of various chemical stimuli is derived from ligand-induced protein conformational transitions, providing critical insights for the design of intelligent biomaterials. Taking calmodulin as an example, it undergoes a conformational shift upon binding specific ligands such as trifluoperazine, transitioning from an extended to a collapsed state, thereby contracting the hydrogel network. In the presence of calcium ions or chlorpromazine, changes in calmodulin’s surface hydrophobicity induce hydrogel swelling [95,96]. This conformational–physical coupling effect is universal; even when calmodulin is employed solely as a network accessory component, the overall material response can be regulated through intramolecular folding [97]. In recent years, novel responsive elements such as repeat-in-toxin (RTX) protein with calcium-dependent folding characteristics and ATP-sensitive adenylate kinase have been developed, further expanding the design dimensions of chemically responsive hydrogels [95,98,99]. These dynamic crosslinking systems achieve cross-scale regulation from microscopic molecular motions to macroscopic material behaviors through the synergistic effects of molecular recognition and conformational transformations, opening new avenues for applications in targeted drug delivery, intelligent microfluidics, and biomimetic microenvironment construction.

#### 3.1.3. Processes Driven by Protein–Protein/Peptide Interaction 

The design strategies for recombinant protein hydrogels have progressively expanded to incorporate specific biomolecular recognition mechanisms. Researchers have explored various natural interaction systems to construct dynamic crosslinking networks through biomimicry. Notably, the complementary recognition between WW domains and proline-rich peptides has been successfully applied in developing two-component injectable hydrogels. By engineering block proteins containing multiple repeated WW domains or ligand peptides, a 3D network exhibiting shear-thinning and self-healing properties can be formed under physiological conditions through simple mixing [100,101]. This dynamic crosslinking system not only supports the 3D culture and directed differentiation of neural stem cells but also provides a precise platform for investigating the influence of microenvironmental mechanical cues on cellular behavior.

In the development of dynamic responsive systems, the ion-sensitive tetratricopeptide repeat (TPR)–DESVD interaction system offers a unique regulatory dimension. By combining TPR proteins with pentapeptide-modified PEG crosslinkers, an intelligent hydrogel can be constructed, whose gel–sol transition behavior is dynamically regulated by the ionic strength of the surrounding solution, adapting to the physicochemical properties of biological fluids [102]. A similar two-component orthogonal assembly strategy includes the Dock-and-Lock system, which leverages the dimerization of kinase subunits and the binding cascade of anchoring proteins to achieve rapid gelation and stepwise enhancement of mechanical properties, thereby providing a carrier that balances injectability and stability for cell delivery applications [103,104].

**Figure 2:**
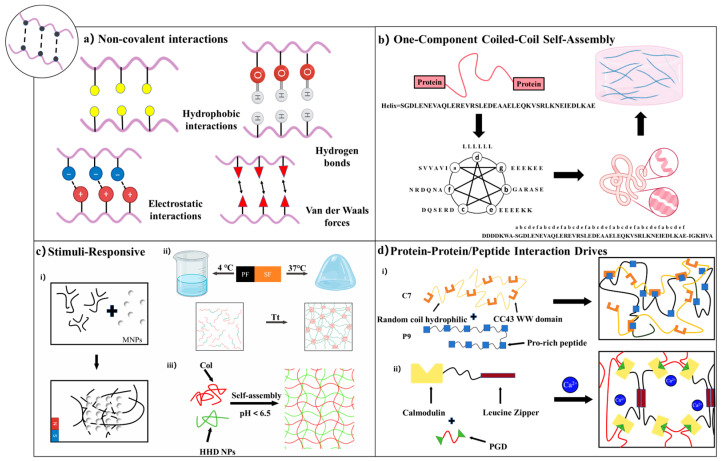
Physical crosslinking strategies for recombinant protein hydrogels [75,85,102]. (**a**) Non-covalent interactions, including hydrophobic interactions, hydrogen bonding, electrostatic forces, and van der Waals interactions. (**b**) One-component coiled-coil self-assembly based on sequence-specific oligomerization of α-helical motifs. (**c**) Stimuli-responsive hydrogels: (**i**) Magnetically responsive hydrogels utilizing embedded MNPs; (**ii**) thermally responsive hydrogels based on conformational changes in SF; (**iii**) photoresponsive hydrogels driven by pH-triggered self-assembly. (**d**) Protein–protein/peptide interaction-driven hydrogels: (**i**) Recognition between WW domains and proline-rich peptide motifs; (**ii**) calmodulin-based hydrogels formed via ligand–protein interaction with leucine zipper motifs.

To further enhance network complexity, multimodal interaction designs have emerged. For instance, integrating the dual mechanisms of calmodulin–ligand recognition and coiled-coil self-assembly enables the construction of hybrid hydrogels with environmental responsiveness. The rheological properties of these hydrogels can be multidimensionally regulated through component selection and environmental stimuli [105]. By precisely tuning protein–peptide affinity and the assembly pathway, these systems open new avenues for constructing tissue engineering scaffolds that combine mechanical robustness with dynamic adaptability (Figure 2).

### 3.2. Chemical Crosslinking Method

Another widely used strategy for preparing protein hydrogels is chemical crosslinking, which involves either the use of multifunctional small-molecule crosslinkers or the incorporation of specialized functional groups into proteins to enable covalent bonding [66,70,74]. Compared to physically crosslinked hydrogels, chemically crosslinked hydrogels typically exhibit greater mechanical strength and stability under physiological conditions due to the formation of permanent covalent bonds, which renders them suitable for diverse biomedical applications [73]. The selection of crosslinking chemistry is often dictated by the presence of particular amino acid residues within the protein matrix. Notably, residues such as tyrosine, lysine, and cysteine offer reactive functional groups (e.g., phenolic hydroxyls, primary amines, and thiols) that can participate in various crosslinking reactions. These residues can undergo chemical modifications or be targeted by specific crosslinkers to form covalent bonds, facilitating the construction of hydrogels with tailored properties. Beyond direct chemical reactions, enzymatic crosslinking has emerged as a compelling alternative, leveraging the specificity and mild reaction conditions of enzymes to catalyze bond formation between protein chains. Enzymes such as transglutaminase, tyrosinase, and horseradish peroxidase (HRP) have been employed to mediate crosslinking reactions involving lysine, tyrosine, and other residues, enabling the formation of hydrogels with controlled architecture and functionality.

In the subsequent subsections, we delve into the predominant chemical crosslinking strategies based on specific amino acid residues and enzymatic methods, highlighting their mechanisms and applications in hydrogel formation.

#### 3.2.1. Chemical Crosslinking Through Side Chains

##### Tyrosine Residue-Mediated Crosslinking

Chemical crosslinking utilizing tyrosine residues offers unique advantages in the design of recombinant protein materials. This approach leverages the oxidative coupling characteristics of the phenolic hydroxyl groups present in tyrosine side chains, enabling covalent bonding within protein networks without the need for exogenous crosslinking agents. For instance, Choi et al. introduced an in situ crosslinking strategy for silk protein hydrogels based on the Fenton reaction. By constructing an Fe^2+^/hydrogen peroxide (H_2_O_2_) redox system, they induced the directional coupling of tyrosine residues in SF. In this system, ferrous ions catalyze the decomposition of H_2_O_2_ to produce highly reactive hydroxyl radicals (·OH), initiating the oxidative coupling of tyrosine phenolic hydroxyl groups between SF molecules, resulting in a dityrosine covalent crosslinking network [106]. This method utilizes the protein’s inherent tyrosine residues as reactive centers, thereby avoiding potential biocompatibility issues associated with heterologous crosslinking agents and preserving the natural conformation and functional domains of the recombinant protein. Building upon this, Qin’s research team developed a photo-driven Fenton reaction catalytic system to achieve controllable crosslinking of recombinant elastin resilin, which is rich in tyrosine residues. By introducing citrate ligands into the reaction system to chelate Fe^2+^, they significantly improved the stability of the photosensitive system and the efficiency of free-radical formation. The photo-Fenton system continuously generates reactive oxygen species (ROS) through an electron transfer mechanism at low concentrations of Fe^2+^, effectively triggering the oxidative coupling of tyrosine residues between resilin molecular chains [40].

In a parallel advancement, Tran et al. investigated a visible light-induced photo-crosslinking method using ruthenium (Ru) and sodium persulfate (SPS) for SF hydrogels. Upon illumination, Ru undergoes photolysis and donates an electron to SPS, forming Ru^3+^, which in turn oxidizes tyrosine residues to generate tyrosyl radicals. These radicals couple to form dityrosine bonds within seconds, reinforcing the hydrogel structure. This strategy not only accelerates gelation but also provides precise spatiotemporal control over hydrogel formation [107]. Collectively, tyrosine-mediated crosslinking represents a versatile and protein-intrinsic strategy for hydrogel fabrication, offering advantages in biocompatibility, network stability, and functional retention.

##### Lysine Residue-Mediated Crosslinking

Crosslinking strategies centered on lysine residues have demonstrated significant advantages in constructing recombinant protein materials, primarily due to the high reactivity and widespread distribution of ε-amino groups on lysine side chains. Lysine residues are abundant on protein surfaces, and their amino groups can undergo specific nucleophilic substitution reactions under mild pH conditions, facilitating controllable intermolecular covalent bonding [108].

Typically, lysine-based chemical crosslinking does not rely on complex oxidation systems or exogenous enzyme catalysis. The crosslinking density can be precisely regulated by adjusting the crosslinking agent’s chain length and reaction time, thereby balancing the mechanical strength and swelling characteristics of the network structure. For example, Zhao et al. developed a two-component bioadhesive by designing a condensation reaction between lysine-rich recombinant ELPs and aldehyde crosslinking agents such as glutaraldehyde (GA) or oxidized hyaluronic acid (OHA). This approach utilizes a high-density lysine-mediated rapid dynamic crosslinking network to achieve second-order curing and ultra-high interfacial adhesion (approximately 101.6 kPa) [109]. Additionally, McGann et al. developed a photo-crosslinked hybrid hydrogel system wherein natural lysine residues in recombinant RLPs serve as ideal sites for chemical modification. Through an amide bond coupling strategy, they achieved controllable functionalization of RLPs, enabling the design of hydrogels with tailored properties for various biomedical applications [110].

Moreover, the dynamic nature of Schiff base linkages formed between lysine amino groups and aldehyde-containing crosslinkers allows for the creation of reversible crosslinking networks. These dynamic covalent bonds confer self-healing properties and responsiveness to environmental stimuli, which are advantageous for applications requiring adaptable material properties. Collectively, lysine residue-mediated crosslinking strategies offer versatile and tunable approaches for engineering protein hydrogels with desirable mechanical properties, biocompatibility, and functional responsiveness.

##### Cysteine Residue-Mediated Crosslinking

Cysteine residues play a pivotal role in the chemical crosslinking of protein hydrogels due to their unique thiol side chains, which can form disulfide bonds under oxidative conditions. Through genetic engineering, the precise introduction of cysteine sites allows for the rational design of crosslinking density and spatial arrangement, enabling accurate regulation of the hydrogel’s topological structure [111]. Tang et al. developed a strategy based on oxidatively triggered disulfide bond formation to achieve protein chain extension and entanglement, which significantly enhances the mechanical properties of the Cys-P_4_-Cys protein hydrogel. Controlled oxidation induces intermolecular disulfide crosslinking, facilitating protein chain extension and the formation of a topologically entangled network. This process substantially improves the structural stability and deformation resistance of the hydrogel. The dynamic entanglement mechanism not only endows the material with exceptional energy dissipation characteristics but also preserves its inherent biocompatibility and injectability [112]. Similarly, Zhou et al. introduced periodic cysteine residues into the molecular design of recombinant SF-ELPs (SELPs), endowing the hydrogel with dynamic crosslinking capabilities. The thermally responsive self-assembly characteristics of SELPs facilitated solution–gel phase transitions at physiological temperatures. By adjusting the structural ratio of SF to elastin, they achieved precise control over the gelation time and mechanical properties of the hydrogel [111].

Photoresponsive crosslinking technology enables precise spatiotemporal control over the crosslinking process by incorporating photosensitive groups or photocatalysts. Unlike traditional chemical crosslinking methods that often require complex external conditions, light-triggered mechanisms can initiate crosslinking reactions directly in targeted areas through specific wavelength illumination. This capability is particularly advantageous for constructing 3D biomaterials and facilitating in vivo applications, where non-invasive and localized control is essential. For example, Farajollahi et al. developed a photocatalytic system based on ruthenium bipyridine complexes that rapidly induced dynamic thiol–disulfide exchange under blue-light excitation, completing network assembly within 30 s while preserving the natural conformation and biological activity of proteins [113].

Moreover, Wu et al. constructed an intelligent wound repair platform by integrating sulfur–silver (S-Ag) dynamic covalent bonds into a recombinant ELP protein hydrogel system [114]. The S-Ag covalent bond acted as a molecular hub, forming a 3D network with mechanical toughness and self-healing properties through dynamic bond cooperation. This design enabled time–space regulation of antibacterial efficiency via programmed silver ion release: during the inflammatory phase, dynamic fracture of the S-Ag bond led to an explosive release of silver ions to eliminate pathogens; during tissue remodeling, it transitioned to a steady-state sustained-release mode, reducing cytotoxicity while maintaining antibacterial activity.

#### 3.2.2. Enzyme-Mediated Crosslinking

Enzyme-mediated crosslinking offers a highly specific and biocompatible strategy for fabricating recombinant protein hydrogels, enabling the construction of complex architectures through selective covalent bonding between targeted amino acid residues [68].

LOX is a copper-dependent amine oxidase that plays a crucial role in the maturation and stabilization of the ECM by catalyzing the oxidative deamination of specific lysine and hydroxylysine (Hyl) residues in collagen and elastin. This enzymatic reaction converts the ε-amino groups of these residues into reactive aldehyde groups, known as allysine and hydroxyallysine, respectively. These aldehyde groups can spontaneously undergo condensation reactions with neighboring amino groups or other aldehyde groups, leading to the formation of various covalent crosslinks, such as Schiff bases and aldol condensates. In collagen, these initial crosslinks can further mature into more-stable trivalent structures like pyridinoline, which are essential for enhancing the mechanical strength and structural integrity of collagen fibrils. The activity of LOX is thus vital for maintaining tissue topology and function. For instance, Yamauchi et al. demonstrated that LOX-mediated crosslinking significantly enhances the mechanical stability of fibrous collagen and preserves tissue integrity [115].

HRP is also widely utilized to catalyze the oxidative coupling of phenolic groups, particularly the tyrosine residues in SF, facilitating the formation of dityrosine bonds that stabilize hydrogel networks [116,117,118]. For instance, Pierantoni et al. developed an SF hydrogel system using HRP and calcium peroxide (CaO_2_), resulting in hydrogels suitable for bone cancer research [117]. Similarly, Partlow et al. employed HRP and H_2_O_2_ to crosslink tyrosine residues in SF, resulting in highly elastic hydrogels with tunable mechanical properties [116]. To augment the phenolic content and thereby improve crosslinking efficiency, Wang et al. introduced carboxyl groups into SF through a carboxylation reaction, producing silk acid (SA) precursors. These were subsequently conjugated with tyramine using an EDC/NHS activation system, yielding SA-TA copolymers with phenolic contents exceeding 7 mol%. Under HRP catalysis, these conjugates exhibited significantly accelerated gelation kinetics, achieving sol–gel transitions within 10 s at physiological temperature [119].

#### 3.2.3. Isopeptide Bond-Based Crosslinking

Isopeptide bond-forming systems, such as SpyTag/SpyCatcher and split inteins, offer innovative strategies for constructing recombinant protein hydrogels. These systems facilitate spontaneous covalent bonding under physiological conditions, providing stable, highly modular platforms for hydrogel formation [120].

In the field of chemically crosslinked hydrogels, the innovation of the SpyCatcher/SpyTag system lies in the deep integration of its spontaneous covalent bond formation mechanism and modular design concept [121]. Derived from splitting the CnaB2 domain of *Streptococcus pyogenes* into a SpyTag peptide and its complementary SpyCatcher fragment, this biomimetic assembly enables rapid, spontaneous reconstitution of isopeptide bonds under physiological conditions [122]. This biomimetic assembly strategy overcomes the stability limitations of traditional non-covalent interactions while preserving the precision of bioorthogonal reactions. Leveraging this approach, researchers have engineered multifunctional tandem protein building blocks that enable on-demand covalent crosslinking of hydrogel networks through the spontaneous formation of isopeptide bonds between SpyTag or SpyCatcher modules. The core advantage lies in the system’s highly modular architecture, allowing for the flexible incorporation of functional elements such as fluorescent markers, cell adhesion motifs (e.g., RGD sequences), and growth factors like leukemia inhibitory factor (LIF) [123,124,125]. This dynamic covalent crosslinking system not only offers excellent biocompatibility and robust encapsulation with sustained fibroblast and stem cell function, but also establishes a chemically programmable platform for precise control of cell fate.

Split inteins, such as the Npu DnaE intein from Nostoc punctiforme, mediate trans-splicing reactions that covalently link protein fragments under physiological conditions. Ramirez et al. engineered a highly stable hydrogel by harnessing the spontaneous trans-splicing activity of a split intein. They designed two block copolymers—CutA-NpuN (N) and NpuC-S-CutA (C)—and employed the DnaE split intein (NpuN/C) from Nostoc punctiforme to catalyze a trans-splicing reaction under physiological conditions. This yielded extended protein chains (J) bearing CutA trimer crosslinking units at both termini, which then self-assembled into a robust hydrogel network. The study further introduced a “stop peptide–stop protein” system, enabling high-density, uniform loading of functional proteins (e.g., green fluorescent protein) by integrating SH3 ligands into the gel matrix [126]. These chemically stable, bioorthogonal hydrogels constitute a versatile modular protein scaffold platform for applications in tissue engineering, biocatalysis, and drug delivery.

**Figure 3:**
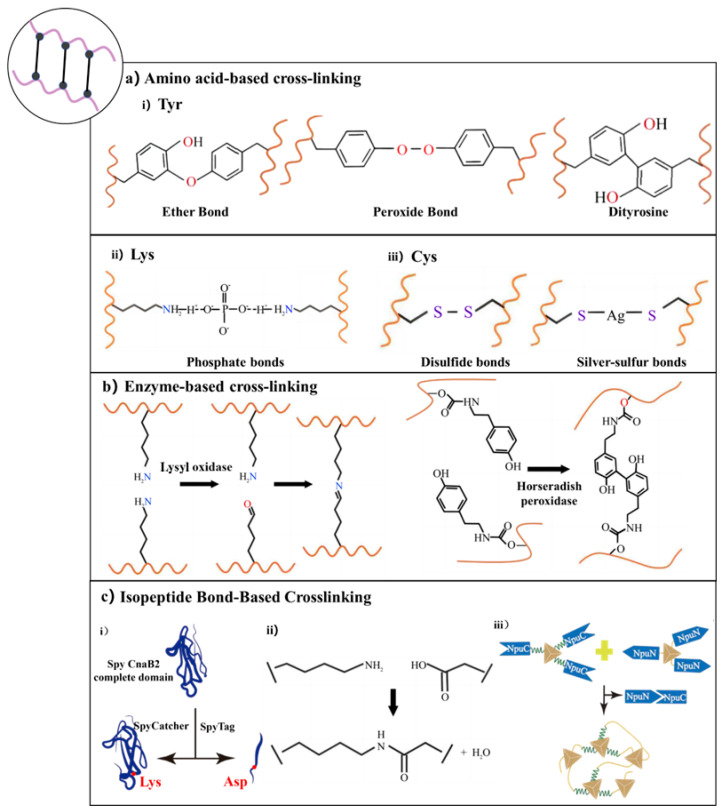
Chemical crosslinking strategies for recombinant protein hydrogels [106,115,121]. (**a**) Amino acid residue-based crosslinking: (**i**) Tyrosine residues form ether, peroxide, and dityrosine bonds via oxidative coupling; (**ii**) lysine residues participate in phosphate-mediated crosslinking; (**iii**) cysteine residues form disulfide or silver–sulfur bonds. (**b**) Enzyme-mediated crosslinking: Crosslinking catalyzed by LOX or HRP, enabling oxidative coupling between specific amino acid residues. (**c**) Isopeptide bond-based crosslinking: (**i**) Covalent bond formation via SpyTag/SpyCatcher recognition system; (**ii**) isopeptide linkage between lysine and aspartate residues; (**iii**) self-assembly of split inteins (e.g., NpuN and NpuC) through trans-splicing reactions.

Each chemical crosslinking strategy, whether leveraging tyrosine oxidative coupling, lysine-driven Schiff base or Michael addition reactions, cysteine-mediated disulfide bonds, enzyme-catalyzed linkages, or isopeptide bond formation, brings unique strengths in terms of reaction specificity, kinetics, and functional versatility (Figure 3). By thoughtfully combining these approaches (for example, integrating enzyme-mediated crosslinks with SpyTag/SpyCatcher modules or supplementing reversible disulfide networks with permanent isopeptide bonds), one can overcome the limitations inherent to any single method. Such hybrid or orthogonal designs enhance mechanical robustness, allow precise spatiotemporal control, and preserve bioactivity—features that are essential for tuning degradation rates, mechanical properties, and cell-instructive cues—ultimately accelerating the translation of hydrogel-based therapies in regenerative medicine [66,70,73].

### 3.3. Interpenetrating Network (IN) System

**Figure 4:**
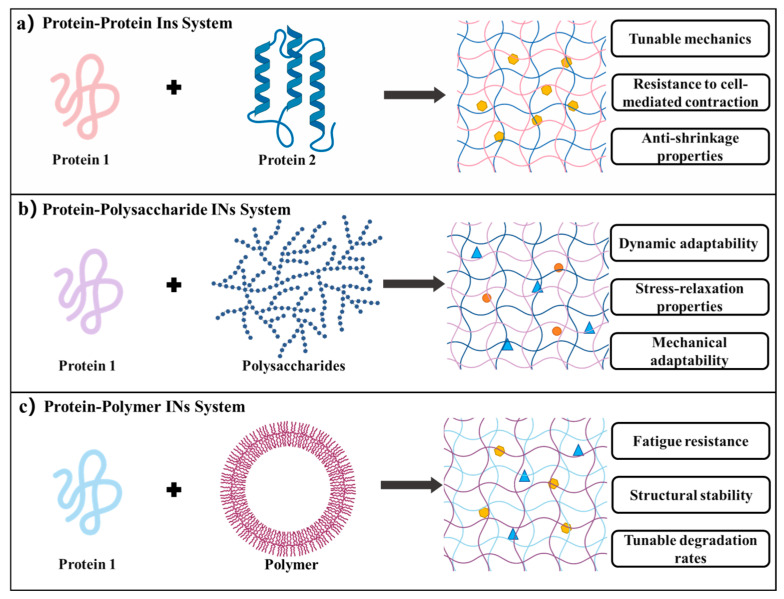
IN hydrogel systems [10,129,136]. (**a**) Protein–protein IN hydrogels: formed by combining two distinct protein networks, offering tunable mechanics, resistance to cell-mediated contraction, and anti-shrinkage properties. (**b**) Protein–polysaccharide IN hydrogels: integrating proteins with polysaccharides to provide dynamic adaptability, stress-relaxation behavior, and enhanced mechanical compliance. (**c**) Protein–polymer IN hydrogels: constructed by incorporating synthetic polymers, characterized by fatigue resistance, structural stability, and adjustable degradation rates.

Recombinant protein hydrogel INs are engineered by integrating two or more independently crosslinked networks, such as protein–protein, protein–polysaccharide, and protein–synthetic polymer systems. By physically or chemically interweaving two or more independently crosslinked networks (without forming covalent bonds between them) an IN achieves mechanical reinforcement, enhanced stability, and multifunctionality that a single network cannot (Figure 4) [127].

#### 3.3.1. Protein–Protein IN System

Protein–protein INs rely on the synergistic crosslinking and dynamic interpenetration of two or more recombinant protein networks. Typically, each protein component is engineered with distinct functional domains that enable sequential or orthogonal crosslinking steps [128]. For example, Ng et al. created a hydrogel IN combining collagen-I and recombinant spider silk protein eADF4 (C16)-RGD. The silk module, genetically modified to include an RGD adhesion motif, forms a rigid nanofibrillar scaffold, while collagen assembles into a dynamic, load-bearing network. Together, they confer tunable mechanics, resistance to cell-mediated contraction (by fibroblasts, myoblasts, and cardiomyocytes), and anti-shrinkage properties [10]. This multi-level assembly preserves each protein’s native features and, via molecular-scale interlocking, enhances overall structural resilience.

Park et al. designed a bilayer IN of gelatin and SF. First, microbial transglutaminase (mTG) catalyzes covalent crosslinks within gelatin. Subsequently, ethanol treatment induces the formation of β-sheet structures in SF, resulting in physical crosslinking that enhances the hydrogel’s mechanical strength and stability. Following freeze-drying and a second mTG step, a multilayer IN emerges. Gelatin’s inherent RGD sequences support cell adhesion and proliferation, while SF’s physical network slows enzymatic degradation by collagenase, aligning scaffold degradation with tissue regeneration timelines. The resulting self-healing, cyclically tolerant IN can withstand prolonged joint motion [129]. Hence, the self-healing capabilities and resilience to cyclic mechanical loading of protein–protein IN hydrogels enable them to withstand the repetitive stresses associated with joint movements, offering a biomimetic solution tailored for the complex mechanical demands of tissue engineering applications.

#### 3.3.2. Protein–Polysaccharide IN System

Protein–polysaccharide INs synergistically enhance biological activity and material properties by sequentially integrating recombinant proteins with functionalized polysaccharides. This approach preserves the specific recognition sites of proteins, ensuring bioactivity, while leveraging the physical crosslinking mechanisms inherent to polysaccharides, such as hydrogen bonding, ionic interactions, and crystallization, to impart tunable mechanical strength and dynamic adaptability to the hydrogel matrix. Such non-covalent interactions not only facilitate environmental responsiveness and reversibility but also contribute to the structural integrity of the hydrogel, making it suitable for applications in tissue engineering and regenerative medicine [130]. For instance, Vorwald et al. developed a fibrin–alginate IN. First, thrombin converts fibrinogen into a fibrous network; then calcium-crosslinked alginate forms a secondary, interpenetrating network. By independently adjusting thrombin and calcium ion concentrations, they precisely controlled the fibrin fiber density, storage modulus, and pore size. This IN maintained cell viability for 14 days and displayed mechanical adaptability as it gradually softened with degradation [131]. This strategy not only preserves the biological functionality of the protein network but also enables precise modulation of swelling behavior, degradation kinetics, and mechanical stability through the tailored topological design of the polysaccharide network. The resulting interpenetrating network addresses the traditional trade-off between mechanical stiffness and bioactivity inherent in single-component hydrogels.

In another example, Zhang et al. devised a fibrin–hyaluronic acid (HA) IN hydrogel system using orthogonal crosslinking. They engineered thiolated HA (HA-SH) and 2-pyridyldithiol-modified HA (HA-PySS) as dynamic crosslinkers: HA-SH mixed with thrombin initiated fibrin polymerization, while HA-PySS participated in thiol–disulfide exchange to concurrently form an HA network. The resulting bicontinuous topology endowed the material with stress-relaxation properties, facilitating cell migration and matrix remodeling [132]. This dynamic microenvironment allows real-time mechanical adaptation in response to cellular forces and prevents structural collapse during long-term culture, making it an ideal carrier for tissue regeneration, drug delivery, and other biomedical applications.

#### 3.3.3. Protein–Polymer IN System

Protein–polymer INs are engineered by integrating two independently crosslinked networks, with one derived from recombinant proteins and the other from synthetic polymers, through either sequential or simultaneous crosslinking processes. In this configuration, the protein network (e.g., SF) imparts biological functionality and provides enzymatic degradation sites, while the synthetic polymer network (e.g., PAAm, PA) contributes fatigue resistance and structural stability. This synergy enables the material to better emulate the complex mechanical demands of native tissue microenvironments. The primary objective is to combine the inherent bioactivity of proteins with the mechanical robustness of polymers without chemically modifying the protein’s active sites [133,134]. For instance, Madaghiele et al. built a PEG–collagen IN via stepwise photo-crosslinking and carbodiimide-mediated chemical crosslinking. Photopolymerized Poly(ethylene glycol) diacrylate(PEGDA)/PA networks provide tunable stiffness, while unmodified collagen retains its cell-adhesive domains. Covalent linkages between collagen and PEG significantly improve mechanical stability and minimize collagen leaching, all while preserving collagen’s dynamic swelling behavior [135].

The hallmark of these INs lies in their ability to achieve a “rigidity–flexibility balance” through multiscale interpenetration, which enables both the retention of protein responsiveness to pH, temperature, or enzymatic cues and a marked improvement in mechanical robustness, self-healing capacity, and environmental adaptability [136]. A prime example is the SF-PAAm (SF-PA) IN. In a single free-radical polymerization step, SF is physically entrapped within a covalently crosslinked PA network. The PA component supplies immediate mechanical support and rapid gelation, whereas SF’s β-sheet domains retain bioactivity. This IN mimics corneal stroma mechanics and significantly boosts corneal stromal cell proliferation, suggesting its potential for ocular tissue regeneration [137]. By combining protein bioactivity with polymer robustness, these IN hydrogels simultaneously overcome the trade-off between mechanical strength and biological function inherent in single-component systems. Such IN systems exhibit enhanced mechanical strength, tunable degradation rates, and responsive behaviors, which are ideal for dynamic drug delivery and load-bearing tissue engineering scaffolds.

The recombinant protein hydrogel IN system achieves dynamic tuning of both biological function and mechanics by molecular-scale interlocking and synergistic crosslinking of multiple networks. Through this multiscale collaboration, IN hydrogels overcome the usual trade-off between mechanical fragility and biological inertia found in single-component gels. Their dynamic, interpenetrating topology imparts stress-dissipating, ECM-like behavior—enabling programmable mechanical gradients, efficient self-healing, cyclic loading tolerance, and cell-guidance cues for tissue regeneration scaffolds. Moreover, because INs preserve each component’s active sites (e.g., protein recognition motifs) while adding polymer- or polysaccharide-derived toughness, they expand recombinant proteins’ utility without requiring genetic modification. IN architectures represent a paradigm shift—from single-function “bionics” to intelligent, stimuli-responsive microenvironments—in regenerative medicine.

Overall, hydrogels that incorporate multiple crosslinking strategies effectively mitigate inherent limitations associated with single-method systems, including but not limited to intramolecular cyclization propensity, crosslinker toxicity, and fabrication complexity. This integrated methodology enables synergistic interactions among distinct crosslinking mechanisms—such as covalent bonds, non-covalent forces, and dynamic networks—which collectively enhance material performance. Consequently, it provides researchers with innovative pathways to transcend conventional material constraints and facilitates the development of next-generation hydrogel platforms characterized by programmable responsiveness, enhanced biocompatibility, and multifunctional adaptability for advanced biomedical applications (Table 1).

## 4. Molecular Engineering Strategy of Recombinant Protein Hydrogels

The molecular engineering strategies for recombinant protein hydrogels employ genetic encoding to rationally design protein sequences, enabling modular assembly of structural domains and directional optimization of functions. The core objective is to balance mechanical properties, dynamic responsiveness, and biofunctional activity [138]. The primary approaches fall into two categories: domain-guided self-assembly design and functional modification via engineered crosslinking sites [139].

### 4.1. Domain-Directed Self-Assembly Design

**Figure 5:**
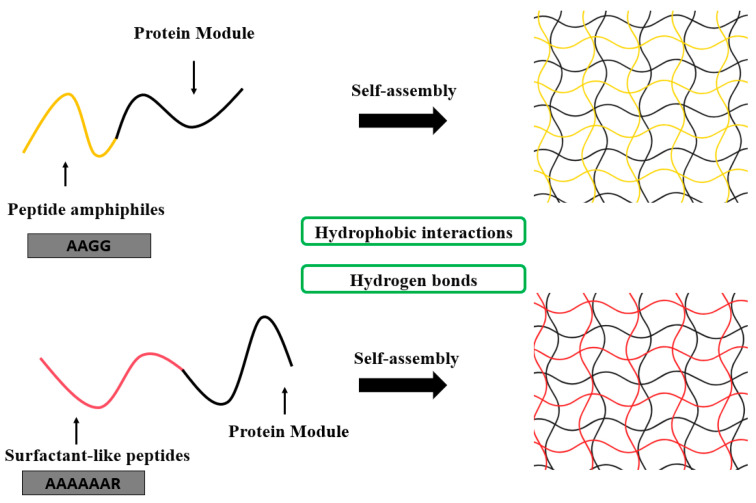
Self-assembled protein hydrogels driven by amphiphilic peptide modules. Hydrogels are formed through the spontaneous self-assembly of proteins containing PA or SLP motifs (e.g., AAGG or AAAAAAR), primarily mediated by hydrophobic interactions and hydrogen bonding, enabling the formation of stable three-dimensional networks [152,154].

Recombinant protein scaffolds with programmable assembly behaviors are constructed by engineering bioinspired modules [8]. Various self-assembly motifs, such as peptide amphiphiles and surfactant-like peptides, facilitate the formation of nanofiber or micellar networks. These networks can be further functionalized with domains like growth factor mimetics and cell adhesion sequences, resulting in hydrogels that mimic the ECM structure while offering tailored bioactivity (Figure 5).

#### 4.1.1. Self-Assembly Module

Recombinant protein self-assembly is a dynamic ordering process coregulated by thermodynamic driving forces and kinetic pathways. Its molecular mechanism arises from the synergistic integration of multiple non-covalent interactions—hydrogen bonds, electrostatic complementarity, and hydrophobic effects [12]. Directional hydrogen bond networks form between amide bonds and carboxyl/hydroxyl groups, while charge balance emerges from electrostatic complementarity between glutamate/aspartate and lysine/arginine residues. Concurrently, hydrophobic association of long alkyl side chains and π-π stacking of aromatic rings further stabilize the assembly [75,76,140]. This multidimensional intermolecular network drives the system toward its lowest free-energy state, yielding supramolecular aggregates with β-sheet or helical conformations [141]. By rationally designing protein sequences, one can precisely tune the energetic barriers of assembly pathways and the topology of the final structures [142]. Moreover, dynamic changes in environmental parameters (e.g., pH, ionic strength, temperature) can induce escape from metastable states, imparting intelligent responsive properties such as self-healing and shear-thinning [143,144,145,146].

Based on their supramolecular architecture, recombinant protein self-assembly systems can be classified into distinct topologies with varied functional characteristics. One common approach fuses peptide amphiphiles (PAs) to recombinant proteins, harnessing peptide amphiphilicity (alternating hydrophilic/hydrophobic regions) for spontaneous nanofiber formation while the protein moiety provides specific bioactivity or structural support (Table 2). The resulting composites combine dynamic physical crosslinking with stable, functionalized networks [147,148,149,150,151]. For instance, Xu et al. genetically engineered PAs (tetrapeptide repeats A_2_G_2_ and V_2_A_2_) fused to vascular endothelial growth factor (VEGF)-mimetic protein (QK) and mitochondrial-targeting peptide (SS31), producing PA1-QK and PA2-SS31 formulations. The fused recombinant proteins drove self-assembly via hydrophobic interactions and hydrogen bonds in the amphiphilic segments, creating nanofiber-based hydrogel networks. In vivo, these hydrogels significantly promoted vascular regeneration, reduced mitochondrial dysfunction and apoptosis, and enhanced cardiac structural and functional recovery [152]. Similarly, Liu et al. generated a bifunctional recombinant protein, pG_EAK, by fusing the Fc-binding domain of protein G (pG) with the amphiphilic self-assembling sequence AEAEAKAK (EAK). When mixed with EAK peptides, pG_EAK formed nanofiber gels that not only assembled via the same amphiphilic driving forces but also retained full IgG-binding affinity. This dual functionality enabled efficient antibody capture, illustrating how self-assembly modules can be tailored for both structural integrity and specific ligand recognition [153].

Surfactant-like peptides (SLPs), characterized by their precisely engineered molecular structures and inherent biocompatibility, also offer a distinct approach to recombinant protein hydrogel design. Mimicking traditional surfactants, SLPs possess an ordered arrangement of hydrophilic headgroups and hydrophobic tails, enabling the spontaneous formation of dynamic nanostructures such as micelles, fibers, or lamellar networks in aqueous solutions. Their amino acid-based composition allows seamless integration with recombinant proteins via genetic fusion, preserving the functional domains of both components. For instance, Chen et al. designed a surfactant-like tetra-tailed amphiphilic recombinant protein, [(C(18))2K]2KR8GRGDS, which self-assembles into hydrogels at low concentrations in aqueous solutions for drug delivery. Through the use of ibuprofen and doxorubicin hydrochloride as model hydrophobic drugs, sustained-release behaviors were observed. By incorporating the RGD peptide sequence for cancer cell targeting and the cell-penetrating octa-arginine (R8) domain, this hydrogel achieves both specific cancer cell recognition and efficient membrane penetration, demonstrating the clinical potential of SLPs in targeted drug delivery [154]. The natural amino acid composition of SLPs reduces immunogenicity, and their degradation products are typically biocompatible small molecules, making them more suitable for in vivo applications compared to synthetic surfactants. In drug delivery scenarios, such hybrid hydrogels not only prolong the local retention of therapeutic proteins but also enable spatiotemporally controlled drug release through microenvironment-responsive mechanisms.

**Table 2:** Self-assembly modules for constructing hydrogels.

Name	Sequence	Class	Reference
EAK16-II	AEAEAKAKAKAEAEAKAK	PA	[155]
RADA16	RADARADARADARADA	PA	[156]
ELK16	LELELKLKLELELKLK	PA	[157]
V2A2D	VVAAD	PA	[158]
V3A3K3	VVVAAAKKK	PA	[159]
V2A2D2	VVAADD	PA	[160]
L1	ARLPRTMVHPKPAQP	PA	[161]
S1	ARLPRTMV	PA	
M1	ARLPR	PA	
(AKKARK)2	AKKARKAKKARK	PA	[162]
G5F	GGGGGF	PA	[163]
G5W	GGGGGW	PA	
SANPA	VVVVKKKKGKKKRAAK	PA	[164]
EV4	EVEV	PA	[165]
R3L12	RRRLLLLLLLLLLLL	SLP	[166]
A6R	AAAAAAR	SLP	[167]
A6D	AAAAAAD	SLP	[168]
V6D	VVVVVVVD	SLP	
G8DD	GGGGGGGGDD	SLP	
K2V6	KKVVVVVVVVV	SLP	
P6K	PPPPPPK	SLP	[169]
P6E	PPPPPPE	SLP	
KP6E	KPPPPPPE	SLP	
APK	AAAAAAPKKPAAAAAA	SLP	[170]
A6K	AAAAAAK	SLP	[171]
V6D2	VVVVVVVDD	SLP	[172]
L6D2	LLLLLLDD	SLP	
R3L12	RRRLLLLLLLLLLLL	SLP	[173]
A3K	AAAK	SLP	[174]

#### 4.1.2. Responsive Modules

**Figure 6:**
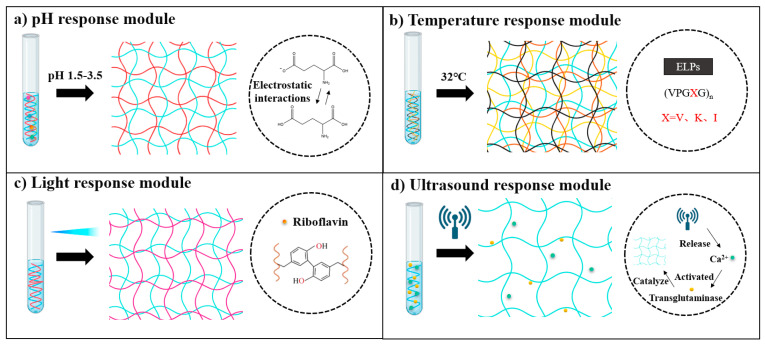
Stimuli-responsive protein hydrogels engineered via functional modules [181,185,186,190]. Illustration of protein hydrogel formation regulated by various external stimuli—(**a**) pH-responsive module: Gelatin-based hydrogels formed via pH-sensitive electrostatic interactions, primarily driven by the protonation or deprotonation of glutamate carboxyl groups. (**b**) Thermal-responsive module: ELPs undergo temperature-induced phase transition governed by specific pentapeptide repeat sequences. (**c**) Photoresponsive module: Light-induced dityrosine crosslinking catalyzed by riboflavin through oxidative activation of tyrosine residues. (**d**) Ultrasound-responsive module: Ultrasound triggers Ca^2+^ release from liposomes, subsequently activating transglutaminase to catalyze fibrin crosslinking and hydrogel formation.

Responsive modules in recombinant protein hydrogels are functional domains engineered at the molecular level to impart dynamic responsiveness to external stimuli such as temperature, pH, enzymes, and light [175,176,177]. These modules regulate the hydrogel’s physicochemical properties, including the crosslinking density, mechanical strength, and degradation rate, or trigger the release of bioactive molecules, enabling intelligent interactions between the material and the biological microenvironment [178,179,180]. Through sophisticated molecular design, these responsive modules confer smart, stimulus-responsive properties to recombinant protein hydrogels (Figure 6) [175].

In pH-responsive systems, researchers have developed precise drug delivery platforms by incorporating proton-sensitive functional groups [179]. For instance, Sun et al. developed a humic acid-based hydrogel that utilizes carboxyl group protonation under gastric acidic conditions (pH 1.5–3.5) to form a dense network, achieving a 98.2% encapsulation efficiency for recombinant porcine interferon fusion protein (rPoIFNα/γ) and significantly reducing pepsin-mediated hydrolysis. Upon the hydrogel’s entry in the intestinal alkaline environment (pH 7.4), deprotonation induces electrostatic repulsion, causing the hydrogel to swell by up to 12-fold and enabling controlled, sustained drug release. This smart delivery system overcomes the biological barrier challenges faced by traditional oral protein therapeutics [181]. Additionally, Mithieux et al. discovered that exposing tropoelastin to alkaline conditions triggers irreversible self-assembly, leading to a sol–gel transition and forming stable, elastic biomaterials without requiring enzymatic or chemical crosslinking [182].

The design of temperature-responsive modules focuses on regulating the phase transition behavior of ELPs [178]. The repeated VPGXG pentapeptide sequence in the ELP molecule imparts unique temperature sensitivity, and precise tuning of the LCST can be achieved by adjusting the ratio of valine to alanine [183]. Meco et al. found that when the temperature exceeded the LCST, PEG-ELP hybrid hydrogels underwent microphase separation, forming an ELP-rich region that resulted in decreased light transmittance. This change in optical properties exhibited more than 95% reversibility over five heating and cooling cycles [184]. In addition, Mizuguchi’s team developed a multifunctional protein hydrogel system with temperature-responsive characteristics through molecular design. The hydrogel achieved performance optimization by integrating four functional peptide modules: temperature-sensitive ELPs endowed the material with controllable sol–gel transition ability at a critical Tt (approximately 32 °C); polyaspartic acid (polyD) chains regulated the aggregation and sustained-release behavior of growth factors through their negative charge characteristics; a de novo-designed helical peptide provided physical crosslinking sites by forming an antiparallel tetramer coiled helical structure; and biological functional peptides were introduced to enhance cell interactions. The study further utilized a bone salivary protein-derived RGD peptide (bRGD) to functionalize the hydrogel, constructing a bRGD-CUBE hydrogel system with pro-angiogenic activity. Additionally, 3D culture experiments showed that human umbilical vein endothelial cells (HUVECs) exhibited significantly enhanced angiogenesis activity in this system. By leveraging the electrostatic interaction between the heparin-binding domain and angiogenic growth factors (such as VEGF and basic fibroblast growth factor (bFGF)), efficient loading and directional release of growth factors were achieved [185].

Light-responsive hydrogels have garnered significant attention due to their capacity for rapid fabrication and functional enhancement through photochemical crosslinking technologies [176]. These hydrogels can be precisely manipulated using light exposure, allowing for spatiotemporal control over their mechanical properties and functionalities. Such capabilities make them highly suitable for various biomedical applications, including tissue engineering, drug delivery, and regenerative medicine. Hemalatha et al. developed an SF–recombinant collagen composite system that forms dityrosine-crosslinked networks via riboflavin-mediated reactions under blue-light irradiation (450 nm, 60 s). This photo-triggered curing mechanism not only enhances the hydrogel’s compressive modulus but also demonstrates significant endothelial repair capabilities. By integrating photomask patterning, the system can create gradient modulus interfaces (15 kPa variation) or microfluidic channels with 50 μm precision, offering programmable solutions for complex tissue regeneration [186]. In contrast to the riboflavin-mediated crosslinking approach, Narayan et al. employed a genetically encoded strategy utilizing SpyTag–SpyCatcher peptide–protein pairs to achieve copolymerization of SELPs with the adenosylcobalamin (AdoB12)-dependent photoreceptor C-terminal domain (CarHC) under mild physiological conditions. This technology enables controlled assembly of two biomacromolecules in biocompatible environments, providing a precise molecular “welding” strategy for constructing light-responsive protein composite hydrogels [187].

In the construction of ultrasound-triggered systems, researchers have overcome the tissue penetration limitations of traditional hydrogels through acousto-enzymatic coupling strategies [188,189,190]. Nele et al. demonstrated that ultrasound-triggered enzymatic catalysis can initiate hydrogelation. In their approach, ultrasound was used to release calcium ions from liposomes, activating transglutaminase catalysis. The ultrasound-activated transglutaminase then catalyzed intermolecular covalent crosslinking between lysine and glutamine side chains of soluble fibrinogen molecules, forming fibrinogen hydrogels. Experiments revealed that calcium ion release, catalytic rates, and hydrogelation kinetics were all dependent on ultrasound exposure duration [191]. Building on this, Zhao’s team proposed a penetration-enhanced ultrasound-triggered system by designing a fibrinogen composite solution containing liposome-encapsulated thrombin and transglutaminase. Under ultrasound irradiation, this system rapidly infiltrates damaged bone tissue and forms stable fibrin networks within 30 s through a cascade reaction (thrombin hydrolyzes fibrinogen→transglutaminase catalyzes crosslinking). Animal experiments confirmed that this strategy significantly promotes microvascular network regeneration, offering an innovative solution to the dual challenges of tissue penetration and rapid hemostasis in trauma repair [192].

### 4.2. Functional Modification Strategies and Biological Activity Regulation

Functionalization strategies empower the intelligent upgrading of hydrogel performance through precise molecular engineering of protein backbones, particularly via site-directed modification of specific amino acid residues [193,194,195]. This molecular-level manipulation enables the incorporation of functionalities such as tissue-targeting capabilities through ligand–receptor interactions, as well as spatiotemporal regulation of mechanical properties and drug release profiles via dynamic covalent and non-covalent bonding. These advances present distinct advantages in the construction of biomimetic tissue interfaces and the development of dynamically regulated drug delivery systems [196,197,198].

#### 4.2.1. Chemical Modification

**Figure 7:**
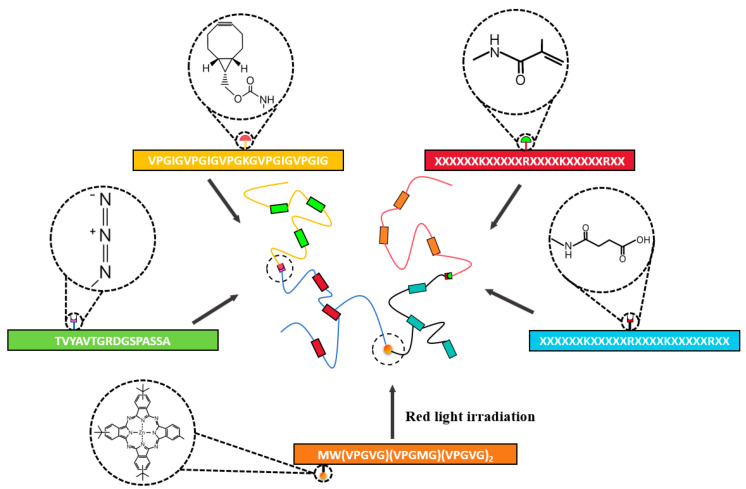
Chemically modified crosslinked protein hydrogels. Hydrogels are formed by chemically modifying specific amino acid residues on proteins using reactive crosslinkers such as PTAD, SA, MA, and PS, enabling covalent network formation through targeted functional group reactions [202,203].

Chemical modification of recombinant proteins offers a versatile molecular engineering approach to enhance and diversify hydrogel functionality. By introducing non-canonical functional groups at specific residues, such as click-chemistry handles, photosensitive moieties, or dynamic covalent linkers, this strategy addresses inherent limitations of natural protein sequences and facilitates multidimensional control over hydrogel properties (Figure 7) [199,200,201].

A key objective is to install reactive handles without perturbing native bioactive sites. For example, Madl et al. employed phenyltriazolinedione (PTAD) to achieve tyrosine-selective modification of ELPs. This method grafted azide groups onto tyrosine side chains while fully preserving lysine ε-amines for subsequent biofunctionalization [202]. Azide-decorated ELPs then underwent strain-promoted alkyne–azide cycloaddition (SPAAC) under mild, aqueous conditions to form 3D hydrogel networks. The extent of azidation directly dictated crosslinking kinetics and mechanical stiffness, whereas unmodified lysines remained available for conjugating cell-adhesive peptides or other ligands, thereby combining orthogonal site specificity with retained programmability and biocompatibility. Similarly, azide–alkyne chemistry has been applied to SF and other recombinant backbones. Introducing vinyl sulfone or alkyne groups into silk proteins creates a multimodal crosslinking platform: Michael addition, enzymatic catalysis, and photoactivation can each trigger gelation and dynamic functional regulation without interfering with cell viability [203]. Such orthogonal chemistries minimize nonspecific side reactions and enable spatial–temporal control over network formation.

Altering surface charge via anhydride chemistry can modulate cell–material interactions. Yang et al. chemically modified collagen hydrogels using methacrylic anhydride (MA) and succinic anhydride (SA) to generate three distinct materials with varied negative charge densities. While preserving the triple-helix structure and comparable storage moduli, these modifications regulated surface hydrophilicity, protein diffusivity, and ligand binding. As negative charge density increased, encapsulated bone marrow mesenchymal stem cells (BMSCs) displayed enhanced glycosaminoglycan (sGAG) production and upregulation of chondrogenic genes (SOX9, COL2A1) via integrin signaling. Thus, anhydride-mediated charge tuning allows simultaneous retention of structural integrity and precise control over cell fate decisions [204].

To impart dynamic responsiveness, non-canonical amino acids or photosensitive moieties can be genetically or chemically introduced. Ibrahimova et al. engineered an ELP scaffold containing methionine residues and conjugated a photosensitizer (PS) via oxidation of methionine. Upon light exposure, PS-generated singlet oxygen (^1^O_2_) oxidized methionine to methionine sulfoxide, altering the hydrophobic–hydrophilic balance and triggering carrier disassembly. This programmable, light-driven disruption enables on-demand release during photodynamic therapy, illustrating how photoresponsive residues can drive spatiotemporal cargo release [205]. Hammer et al. developed degradable carbamate linkers to achieve reversible protein–polymer conjugation. By fine-tuning benzene substituents on the carbamate, they controlled hydrolysis rates and thereby regulated the dissociation kinetics of lysozyme–PEG complexes. When formulated as a hydrogel, roughly 60% of lysozyme was released over 7–21 days, with the enzyme’s secondary structure and activity preserved throughout. Such degradable linkers exemplify how electronic modulation at the linker level can yield tunable release profiles while maintaining protein function [206].

Chemical modification enables precise tuning of recombinant protein hydrogels, extending their functional scope beyond native limitations and introducing dynamic, environment-responsive behaviors. However, key challenges, such as site selectivity, retention of biocompatibility, and scalable implementation, still hinder broader application. Moving forward, the synergy between molecular engineering and bioorthogonal chemistry will be crucial for creating clinically relevant, next-generation smart biomaterials.

#### 4.2.2. Functional Module Integration

**Figure 8:**
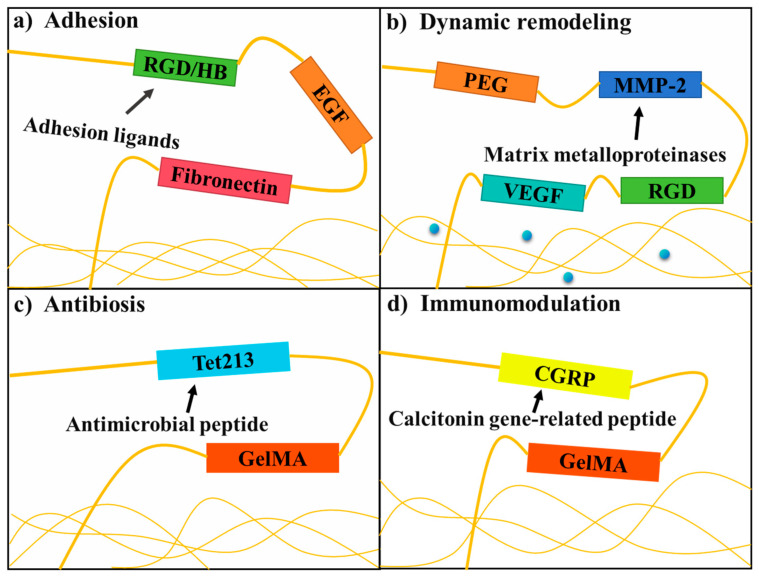
Functional protein hydrogels [207,209,212,214]. (**a**) Integrin-binding motifs (RGD/HB) combined with growth factors (EGF) promote cell adhesion and tissue-specific functions; (**b**) MMP-cleavable sequences allow cell-responsive matrix degradation, while integrated growth factors (VEGF/TGF-β1) guide tissue regeneration; (**c**) conjugated AMPs exhibit direct bactericidal effects; (**d**) bioactive peptides (CGRP/laminin) drive macrophage polarization toward regenerative M2 phenotypes while stimulating angiogenesis.

Functional module integration capitalizes on the modular design of recombinant proteins to not only recapitulate but also expand the dynamic signaling repertoire of the ECM. By embedding short peptide motifs directly into the primary sequence or by chemoselectively conjugating bioactive ligands post-synthesis, these hydrogels can present spatiotemporally controlled signals that regulate cell adhesion, proliferation, migration, and differentiation (Figure 8).

In one strategy, adhesion ligands such as RGD or HB sequences are genetically fused to a self-assembling β-sheet peptide backbone, enabling integrin engagement that clusters focal adhesion kinase (FAK) and activates downstream signaling. For instance, Mehta et al. employed self-assembling peptide nanofiber gels as modular scaffolds to explore the synergistic regulation of hepatocyte behavior by fibronectin-derived ligands (RGD and HB) and epidermal growth factor (EGF). Their study demonstrated that co-presentation of RGD and HB motifs significantly enhanced hepatocyte aggregation, spreading, and metabolic activity in the presence of soluble EGF stimulation, while immobilized EGF promoted further proliferation and DNA synthesis across all ligand conditions [207]. Similarly, Cambria et al. used sortase-mediated transpeptidation to site-specifically graft human EGF onto PEG hydrogels. Incorporating the sortase recognition sequence (LPRTG) into the hydrogel network enabled enzymatic conjugation of GGG-EGF via its N-terminal GGG motif. The immobilized EGF retained full biological activity and demonstrated a direct correlation between conjugation density and cell proliferation in primary hepatocytes and endometrial epithelial cells [208].

Beyond static presentation, enzyme-responsive linkers introduce dynamic remodeling. For example, Seliktar’s group developed a matrix metalloproteinase-2 (MMP-2)-sensitive PEG hydrogel with dual functionalities by integrating RGD motifs and engineered VEGF. Crosslinking via MMP-cleavable peptide sequences enabled cell-mediated degradation, while VEGF and RGD cofunctionalization promoted endothelial cell migration, activation, and progressive hydrogel remodeling. The addition of transforming growth factor β1 (TGF-β1) further upregulated MMP-2 activation, enhancing degradation and enabling spatiotemporally regulated angiogenesis [209]. Similarly, Zhang et al. engineered an MMP-2-responsive peptide hydrogel designed for the breast tumor microenvironment. This hydrogel integrates photothermal agents and immune checkpoint inhibitors, enabling controlled degradation and release of therapeutic agents in response to MMP-2 activity. The system effectively modulates the tumor immune microenvironment, enhancing antitumor efficacy through synergistic multimodal therapy [210].

Recombinant protein hydrogels integrated with antimicrobial peptides (AMPs) or proteins demonstrate significant promise for combating infections while promoting tissue repair. For instance, Xie and colleagues developed an antibacterial hydrogel by grafting the antimicrobial peptide Tet213 onto gelatin methacryloyl (GelMA) via thiol–ene click chemistry. In vitro studies demonstrated that this modified composite hydrogel effectively eliminated dental pulp microbiota, alleviated inflammatory responses in human dental pulp stem cells (hDPSCs), and promoted odontogenic differentiation through activation of the peroxisome proliferator-activated receptor gamma (PPARγ) pathway [211]. Pushing the boundaries of multifunctional design, Wang’s team engineered a hydrogel dressing incorporating Cypate-conjugated AMPs, which confer triple antibacterial action through intrinsic antimicrobial activity, photothermal therapy (PTT), and photodynamic therapy (PDT). To further enhance PDT efficacy and modulate hypoxia, the system includes a lipid-encapsulated oxygen carrier (perfluorodecalin). Additionally, recombinant type III collagen is integrated to provide structural support and promote tissue regeneration. This synergistic platform not only eradicates pathogens but also accelerates wound healing by enhancing oxygenation and re-epithelialization [212]. Beyond AMPs, the incorporation of nonpeptide antimicrobial proteins offers additional functionalities. For instance, Sundaran et al. developed a novel hydrogel by functionalizing SF with the broad-spectrum antimicrobial peptide ε-poly-L-lysine (EPL). The resulting SF-EPL hydrogel exhibited strong antimicrobial activity against major pathogens, including Pseudomonas aeruginosa, Klebsiella pneumoniae, and methicillin-resistant *Staphylococcus aureus* (MRSA). Notably, beyond its antimicrobial properties, the engineered hydrogel also modulated the wound microenvironment by scavenging hydroxyl radicals, thereby exerting antioxidant effects, and neutralizing lipopolysaccharides (LPSs), contributing to anti-inflammatory activity [213].

Engineered domain conjugation also imparts immunomodulatory and regenerative functionalities to recombinant protein hydrogels. Li and colleagues developed a calcitonin gene-related peptide (CGRP)-modified GelMA hydrogel, demonstrating excellent biocompatibility and immunomodulatory properties. In vitro studies revealed that CGRP promotes macrophage polarization toward the reparative M2 phenotype through suppression of the p53 signaling pathway and enhances endothelial proliferation to facilitate angiogenesis [214]. Building on the concept of employing bioactive peptides to orchestrate regenerative niches, Nam et al. developed a laminin-111-derived peptide-modified fibrin hydrogel (L1P-FH) tailored for salivary gland repair. This hydrogel was constructed by covalently conjugating two key functional peptide motifs (A99 and YIGSR), mimicking the bioactivity of native laminin. L1P-FH not only facilitated the structural regeneration of glandular tissue but also enhanced the expression of angiogenic markers and neurogenic proteins, thereby reconstructing a functional glandular microenvironment. Importantly, the hydrogel exhibited favorable immunomodulatory effects, controlled biodegradability, and excellent biocompatibility in vivo [215].

The integration of functional modules into recombinant protein hydrogels leverages the programmability of molecular design to replicate and amplify complex extracellular cues. By incorporating adhesion motifs (e.g., RGD, HB), growth factors (e.g., EGF, VEGF), enzyme-responsive linkers, and therapeutic agents (e.g., AMPs, immune modulators), these hydrogels achieve spatiotemporally regulated cell-instructive signaling, dynamic degradation, antimicrobial action, and immunomodulatory control. Strategies such as genetic fusion, enzymatic ligation, and site-specific chemical modification allow for precise spatial patterning and synergistic functionality. These advances collectively establish recombinant protein hydrogels as versatile and tunable platforms that surpass traditional materials in structural mimicry, bioactivity, and adaptive responsiveness. As the understanding of multiscale assembly mechanisms deepens, this biomaterial system holds substantial promise for precision tissue engineering, regenerative medicine, and smart therapeutic delivery.

Although individual molecule engineering strategies significantly enhance hydrogel performance, they each exhibit inherent limitations—such as insufficient mechanical strength, potential biotoxicity, and limited environmental responsiveness—that hinder practical applications (Table 3). By synergistically combining different molecular engineering strategies, the performance bottlenecks associated with monolithic systems can be effectively addressed. This convergent design paradigm not only broadens the functional landscape of recombinant protein hydrogels but also opens up new avenues for innovation in biomedical material research.

## 5. Application Direction of Recombinant Protein Hydrogel

Leveraging their molecular programmability and capacity for functional integration, recombinant protein hydrogels are catalyzing a paradigm shift in biomedical engineering toward precision, intelligence, and adaptability. Through genetic engineering and biomimetic design, these materials enable dynamic modulation of mechanical properties, degradation kinetics, and bioactivity [216,217,218,219,220,221]. They establish a synergistic intervention framework across the “material–cell–microenvironment” axis, addressing complex clinical needs in tissue regeneration, drug delivery, bone engineering, and emerging frontier applications (Table 4).

### 5.1. Tissue Engineering

Recombinant protein hydrogels, with their biomimetic architecture and precisely engineered biological functionalities, are redefining the technological landscape of tissue engineering [231,232,233,234,235,236]. Through advanced molecular engineering strategies, multifunctional hydrogel systems have been developed for diverse biomedical scenarios including wound healing, cell therapy, and ocular disease management [237,238,239,240].

Effective skin regeneration demands hydrogels with antimicrobial activity, pro-healing capabilities, and tunable mechanical performance. Traditional dressings often fail to reconcile the dynamic demands of the wound microenvironment with sustained therapeutic efficacy. In contrast, next-generation protein hydrogels offer integrated solutions via hierarchical biomimetic design. For example, Zhang et al. developed an SF/sericin composite hydrogel inspired by silkworm cocoon architecture, with 1,2-dimyristoyl-sn-glycero-3-phosphoglycerol (DMPG) as a physical crosslinker, to form an injectable, self-healing network. This system exhibits rapid shear-thinning recovery and achieves 99.9% antibacterial efficacy against *Staphylococcus aureus* and *E. coli*. In vivo studies revealed accelerated wound closure (~40% increase) and significantly reduced IL-6 levels (~35% of control), highlighting its promise for both acute and chronic wound management [4]. At the same time, Li et al. developed a rationally designed recombinant collagen-like protein (RC) incorporating multiple bioactive motifs. Through the use of EDC/NHS-mediated crosslinking, the purified RC was conjugated with GelMA to generate GelMA-RC hydrogels. In vivo experiments demonstrated enhanced wound-healing efficacy, primarily due to upregulated expression of cytokeratin-14 and collagen type I alpha (Col1α) [220]. Meanwhile, Xu’s team addressed the rapid degradation of extracellular vesicles (EVs) in wound sites by using transglutaminase to covalently crosslink recombinant human type III collagen (rhCol III) with EVs, forming a sustained-release hydrogel. This design enhanced M2 macrophage polarization, fibroblast migration, and endothelial angiogenesis. In diabetic wound models, the hydrogel promoted tissue repair by suppressing inflammation and stimulating vascularization [241]. To address the challenge of skin regeneration, Hu et al. developed a composite hydrogel integrating sodium alginate@MnO_2_ nanoparticles, recombinant humanized type III collagen (RHC), and mesenchymal stem cells (MSCs). In diabetic wound models, this multicomponent hydrogel significantly accelerated healing, promoting rapid re-epithelialization, enhanced collagen deposition, and robust neovascularization [221]. These studies collectively highlight that protein hydrogels, through hierarchical biomimetic design and strategic integration of bioactive components, enable simultaneous modulation of mechanical properties, antimicrobial activity, and spatiotemporal delivery of regenerative signals. This multifaceted functionality overcomes the inherent limitations of conventional wound dressings, positioning recombinant protein hydrogels as next-generation smart biomaterials with both adaptive responsiveness and therapeutic precision for the effective treatment of acute and chronic wounds.

On the other hand, scar formation following skin injury remains a significant challenge in regenerative medicine. Various wound dressings composed of recombinant protein alone or combined with bioactive factors have been developed to improve healing outcomes. Drawing inspiration from the structural, compositional, and biological features of native skin, Zhang et al. engineered a hydrogel incorporating modified recombinant human type III collagen and thiolated hyaluronic acid to promote skin appendage regeneration and improve post-injury functional recovery by minimizing fibrotic scarring. The hydrogel exhibited excellent biocompatibility, antioxidant capacity, and pro-angiogenic activity and stimulated fibroblast migration in vitro. In a rat full-thickness wound model, it suppressed inflammation, promoted microvascular formation, and significantly accelerated wound closure [242]. Similarly, Yan et al. designed a recombinant neurotrophin-3 (NT3) fused with the silk fibroin light chain (SFL), yielding an NT3-functionalized SF hydrogel. The SFL-NT3 fusion protein bound effectively to the heavy and light chains of silk fibroin and was efficiently incorporated into the hydrogel matrix. This hydrogel not only accelerated wound healing but also upregulated type III collagen (Col3) expression and induced folliculogenesis during tissue regeneration—key features associated with scarless wound healing [224].

Skin aging, a complex physiological process characterized by collagen degradation, presents substantial dermatological challenges. Wang et al. developed a highly bioactive hydrogel implant using tetrakis(hydroxymethyl)phosphonium chloride (THPC) as a crosslinker for recombinant collagen to promote skin rejuvenation. Compared to conventional crosslinkers such as EDC/NHS and BDDE, THPC demonstrated superior crosslinking efficiency, achieving complete recombinant collagen network formation at minimal concentrations. Its tetrafunctional reactivity facilitated robust interactions with triple-helical recombinant collagen, resulting in hydrogels with enhanced mechanical strength, injectability, stability, and long-term durability. The resulting hydrogel significantly promoted the proliferation, adhesion, and migration of human foreskin fibroblast-1 (HFF-1) cells. In vivo, it improved dermal density and skin elasticity while reducing transepidermal water loss, thereby creating a favorable microenvironment for fibroblast activity and the regeneration of healthy collagen [225].

Clinical cell therapies are often hindered by low cell survival and poor integration with host tissue microenvironments. Recombinant protein hydrogels, featuring programmable stiffness and controlled degradation, can serve as ideal carriers for cell transplantation. Gregorio et al. developed a hydrogel based on XTEN and coiled-coil P modules from rat cartilage oligomeric matrix protein, enabling injectable networks with tunable stiffness (0.1–10 kPa) and >95% cell viability over 72 h post-injection [243]. Integration of a photocleavable protein (PhoCl) further allowed on-demand degradation upon 405 nm light exposure. This smart delivery platform extended Chimeric Antigen Receptor T-Cell Immunotherapy (CAR-T) cell residence in tumor tissues from 3 to 14 days, offering a novel approach to minimize systemic toxicity while enhancing local efficacy [244]. Huang et al. also addressed challenges in mesenchymal stem cell (MSC) therapy for peripheral artery disease (PAD) by designing a Nap-GFFYK–thiol hydrogel based on dynamic disulfide exchange. This ECM-mimetic network enabled responsive degradation and sustained release of bioactive peptides. In murine models of hindlimb ischemia, the hydrogel significantly improved hP-MSC retention, reduced inflammation, enhanced angiogenesis, and delayed fibrosis [245]. The integration of dynamic crosslinking networks with stimulus-responsive modules enables protein hydrogels to closely emulate the mechanical and biochemical characteristics of the ECM. These intelligent carriers markedly enhance the survival and functional retention of transplanted cells, while further amplifying therapeutic efficacy through precise, spatiotemporally controlled delivery of bioactive cues. As such, they offer essential technological support for advanced applications in CAR-T-cell therapy and stem cell-based regenerative medicine.

Chronic management of posterior eye diseases faces challenges such as short drug half-lives and risks associated with frequent intraocular injections. Recombinant protein hydrogels, with customizable release profiles and excellent ocular biocompatibility, provide a promising solution. Yaylaci et al. developed a β-sheet nanofiber peptide hydrogel for sustained anti-VEGF delivery. By encapsulating bevacizumab within the nanoscale fiber network (20–50 nm), they created an intraocular depot that extended drug bioactivity from 4 to 12 weeks. In rabbit models, this system reduced choroidal neovascularization by 78% without adverse effects such as elevated intraocular pressure or vitreous opacity [246]. By synergistically modulating physical entrapment and chemical interactions, this self-assembled nanofiber-based delivery system enables sustained release of macromolecular therapeutics within the vitreous. This strategy significantly prolongs the therapeutic window while minimizing the need for invasive interventions, offering a promising solution for the precise treatment of chronic ocular diseases such as age-related macular degeneration.

### 5.2. Drug Delivery Systems

The core advantage of recombinant protein hydrogels in drug delivery lies in their highly programmable drug loading and release mechanisms. By integrating physical entrapment, chemical conjugation, dynamic crosslinking, and environmentally responsive modules, researchers can precisely control drug encapsulation efficiency, release kinetics, and targeting specificity. These strategies provide multimodal solutions for precision therapy in complex pathological microenvironments [247,248,249,250].

Achieving precise spatiotemporal control over drug release remains a central challenge for optimizing therapeutic efficacy. Design approaches such as aptamer functionalization and live cell encapsulation enable intelligent modulation of drug kinetics via molecular recognition and sustained bioactivity. For example, Zhao and colleagues engineered an aptamer-functionalized fibrin hydrogel for the in situ delivery of VEGF and PDGF-BB. By covalently linking specific aptamers to fibrinogen, the resulting injectable hydrogel exhibited a hierarchically porous structure. In vivo studies demonstrated that this system achieved coordinated, temporally regulated release of both growth factors, significantly enhancing angiogenesis in animal models [251]. Arndt et al. reported a hydrogel system derived from recombinant spider silk protein NT2RepCT, which forms at 37 °C. The mechanical strength and diffusion properties could be modulated by protein concentration. When ARPE-19 cells were encapsulated within these hydrogels, secretion of human progranulin (PGRN) was sustained for over 31 days, with increasing concentrations detected over time—demonstrating minimal cytotoxicity and the feasibility of long-term protein delivery [226]. In addition, to overcome delivery challenges, Xu et al. developed a hydrogel delivery system based on polylysine-modified apoferritin. This carrier was engineered by genetically modifying four lysine residues at the N-terminus of apoferritin to introduce polylysine chains, enabling efficient loading and delivery of Sirtuin1-encoding saRNA to chondrocytes. The key mechanism involves the polylysine modification imparting a “proton sponge effect,” which facilitates lysosomal escape; concurrently, the carrier undergoes pH-sensitive degradation in acidic environments, thereby achieving controlled saRNA release. This approach effectively inhibits chondrocyte apoptosis, enhances cell migratory capacity, improves cartilage protection, and ultimately alleviates osteoarthritis symptoms in vivo [227]. Aptamer-functionalized networks enable selective and temporally controlled release of bioactive factors, while recombinant protein-based encapsulation offers a platform for sustained delivery of therapeutic cell secretions. Together, these strategies overcome the limitations of single-mode release systems and open up new opportunities for angiogenesis and cell therapy.

Light-responsive drug release technologies offer non-invasive, highly localized control, yet their clinical translation is hampered by limited tissue penetration and drug loading efficiency. Photoresponsive recombinant protein hydrogels present an innovative solution. Wang et al. developed a triblock protein polymer (CEC) composed of elastin (E) and coiled-coil (C) domains, crosslinked by NHS–diazirine (D) to form a photopolymerizable hydrogel (CEC-D) [252]. Experimental results demonstrated that the resulting hydrogels exhibited high encapsulation efficiency and enabled controlled release of curcumin into the supernatant, thereby achieving effective drug delivery. Moreover, by employing photopatterning techniques to fabricate microscale stripe patterns on the hydrogel surface, the release profiles could be precisely modulated and optimized [253]. Song et al. synthesized protein hydrogels (K180L20 and E180L20) that self-assemble in vivo upon intracerebral injection [254]. The mechanical properties of these hydrogels could be finely tuned by adjusting the salt concentration in the diluent or by incorporating proteins. Moreover, these hydrogels enabled the sustained in vivo release of the neurotrophic factor NGF across the blood–brain barrier. The released bioactive proteins exerted localized cellular effects over prolonged subacute periods, with diffusion distances extending several millimeters [208]. Through the coupling of photo-crosslinkable hydrogels with micropatterning, drug release profiles can be dynamically modulated via network density and topological control. This “photopatterning–release” strategy enhances delivery precision, especially in deep-tissue targets such as the brain.

Despite the inherent biocompatibility and responsiveness of these hydrogels, achieving a balance between stable drug loading and on-demand release still demands molecular-level innovation. Xu’s group addressed this challenge by designing a recombinant hydrogel system based on tetrameric self-assembly of two engineered proteins, ULD-TIP1 and ULD-GGGWRESAI. Through specific non-covalent interactions, these proteins rapidly form a 3D, hierarchically porous network. The resulting hydrogel supports diffusion-driven rapid release of hydrophilic drugs like diclofenac (DIC) within 24 h, while showing a minimal inflammatory response during in vivo degradation, confirming its biosafety and long-term applicability [255]. Akrami’s team also developed a composite delivery system by embedding dexamethasone-loaded chitosan nanoparticles into SF hydrogels (SFHs) via ionic crosslinking. This hierarchical platform extended local drug retention to 16 days, demonstrating great potential for anti-inflammatory therapies [256,257]. Non-covalently assembled hydrogels enable differential release of hydrophilic and hydrophobic drugs through engineered pore structures and nanocomposite integration. This dual-function “intelligent loading–responsive release” strategy enhances both local drug bioavailability and therapeutic precision, offering a new paradigm for managing inflammatory microenvironments.

Beyond bulk hydrogels, recombinant protein-based nanogels and microgels offer distinct advantages for drug delivery. Nanogels, characterized by their nanoscale crosslinked networks and high surface-area-to-volume ratio, readily penetrate biological barriers such as the blood–brain barrier, making them particularly suitable for targeted drug delivery and molecular imaging applications [258]. For instance, Rather et al. developed an injectable ELP nanogel (ENG) for efficient delivery of Decursin—a small-molecule inhibitor blocking the Wnt/β-catenin pathway in prostate cancer. Decursin–ENG exhibited moderate cytotoxicity against DU145 cells, balancing efficacy and safety, and leveraged the enhanced permeability and retention effect to concentrate the drug at tumor sites more effectively than free Decursin [259]. In contrast, microgels form micrometer-scale, 3D networks with pronounced swelling and deswelling, providing prolonged local retention ideal for cell scaffolding, sustained macromolecule release, and bioprinting [260]. For example, Chen et al. engineered methacrylate-crosslinked HA microgels conjugated to celecoxib via an MMP-2-responsive peptide linker (GGPLGLAGGC) and a collagen-binding peptide (WYRGRL). In osteoarthritis conditions, elevated MMP-2 cleaves the linker to trigger on-demand drug release, suppressing macrophage activation, reducing pro-inflammatory cytokines, and protecting chondrocytes. The collagen-binding peptide further anchors the microgels to cartilage, forming a lubricating layer that diminishes wear [228].

From molecular-recognition-based release to light-triggered spatiotemporal programming, recombinant protein hydrogels are emerging as “smart drug factories” with multidimensional regulatory capabilities [248,261]. These innovations not only overcome the limitations of traditional drug delivery systems, such as low drug loading capacity and imprecise release, but also pave the way for single-dose, long-acting precision therapies through dynamic interactions among materials, drugs, and pathological environments [246]. With the continued integration of cross-scale manufacturing techniques and AI-driven prediction models, recombinant protein hydrogels are poised for intelligent, end-to-end upgrades in next-generation drug delivery systems.

### 5.3. Bone Regeneration Engineering

The deep integration of recombinant protein hydrogels with bioprinting technologies is redefining the frontiers of tissue engineering innovation [262,263,264,265,266,267]. Through the multidimensional synergy of gene editing, molecular biomimetics, and intelligent material design, these “bioinks” enable the precise integration of biomimetic tissue architecture, tunable mechanical properties, and spatially controlled bioactivity. This establishes a foundational platform for the construction of complex, functional organ prototypes [268,269,270,271,272,273,274,275]. This section focuses on bone and cartilage regeneration, highlighting how recombinant protein hydrogels overcome the limitations of traditional tissue engineering via cross-scale fabrication strategies.

Personalized bone defect repair demands a dynamic balance between mechanical support, biodegradation, and osteoinductive activity. Traditional bone substitutes often fail to simultaneously achieve anatomical conformity, osteointegration, and controlled release of growth factors. In contrast, recombinant protein hydrogels, when combined with precision manufacturing, offer a systematic solution. For example, Wagner et al. employed collagen-based hydrogels as carriers for recombinant human bone morphogenetic protein-2 (rhBMP-2) to explore its regulatory role in bone regeneration and scaffold-mediated bone formation. Using 3D image reconstruction and computer-aided design/manufacturing (CAD/CAM) technologies, they developed a personalized mandibular scaffold composed of a permanent zirconia (ZrO_2_) framework for mechanical strength and β-tricalcium phosphate (β-TCP) filler to promote osteointegration. In vivo studies in a Göttingen minipig model showed significant osteogenic outcomes with the rhBMP-2-loaded ZrO_2_-β-TCP scaffold [276]. He et al. developed an injectable hydrogel system for cranial bone regeneration based on triple-helical recombinant collagen (THRC), integrating bone morphogenetic protein-2 (BMP-2) through molecular design and biomimetic strategies. This hydrogel formed in situ within 2 min under physiological conditions via Schiff base reactions among oxidized carboxymethyl cellulose (OCMC), THRC, and N-succinyl chitosan (NSC). The resulting 3D network mimicked bone matrix topologies and exhibited excellent biocompatibility and bioactivity, significantly promoting cell proliferation, adhesion, and differentiation and new bone formation [277]. Although basic fibroblast growth factor (bFGF) significantly promotes bone repair, its instability under physiological conditions limits its therapeutic efficacy. To address this, Guo et al. designed a novel recombinant human collagen (rhCol) capable of enzymatic crosslinking via transglutaminase (TG) to form rhCol/bFGF hydrogels. The resulting porous hydrogel exhibited favorable mechanical strength and effectively supported cell proliferation, migration, and adhesion. Importantly, its controlled degradation profile enabled sustained release of bFGF, thereby enhancing its bioavailability and osteoinductive potential. In a rat calvarial defect model, application of the rhCol/bFGF hydrogel significantly accelerated bone regeneration, demonstrating its promise for clinical bone repair applications [230]. These examples from CAD/CAM-customized composite scaffolds to injectable hydrogels demonstrate how recombinant protein hydrogels enable precise coupling of mechanical gradients with temporally controlled bioactivity. This “structure–function” dual-driving strategy offers comprehensive support for personalized bone regeneration, from macroscopic structural adaptation to microscopic cellular regulation.

Cartilage regeneration poses unique challenges due to its avascular nature and low metabolic activity, necessitating materials that provide long-term mechanical stability and dynamic microenvironmental cues. Recombinant protein hydrogels meet these requirements through biomimetic signaling and physically triggered crosslinking mechanisms. Lamparelli et al. created a 3D biomimetic cartilage regeneration system by embedding poly(lactic-co-glycolic acid) microcarriers (PLGA-MCs) loaded with human transforming growth factor-β1 (hTGF-β1) into a collagen matrix. The PLGA-MCs, fabricated via supercritical emulsion extraction, exhibited controlled release over 21 days. This 3D system, combined with perfusion-driven mass transport, effectively induced chondrogenic differentiation of human bone marrow mesenchymal stem cells (hBM-MSCs), providing a robust platform for in vitro cartilage engineering and mechanistic studies [278,279,280]. Wu et al. designed a self-assembling peptide hydrogel (Ac-LIANAKGFEFEFKFK-NH_2_, LKP) that mimics TGF-β1 signaling. The peptide formed nanofiber networks (8~12 nm) via β-sheet-driven assembly and showed strong chondroinductive activity in vitro. They further combined LKP with glycidyl methacrylate-modified SF (SF-GMA) to create a photo-crosslinkable injectable composite scaffold. This hybrid system enhanced compressive strength through SF-GMA polymerization while maintaining excellent cytocompatibility. In vivo studies confirmed that 10–20% LKP-containing scaffolds (SF-GMA/LKP10, SF-GMA/LKP20) effectively repaired osteochondral defects in rabbits [281,282,283]. Yuan’s team introduced an ultrasound-triggered SF hydrogel system for cartilage regeneration. High-frequency ultrasound induced β-sheet transitions in SF chains, enabling rapid, chemical-free gelation. Hydrogels prepared at 50% ultrasound amplitude exhibited cartilage-matching mechanical properties and slow degradation, facilitating cell adhesion and proliferation and tissue remodeling. Subcutaneous implantation in nude mice and in situ injection into rabbit cartilage defects confirmed their robust in vivo regenerative potential [284]. These cutting-edge strategies, including spatiotemporally controlled microcarriers, functional self-assembling peptides, and ultrasound-triggered gelation, successfully mimic the dynamic biomechanical and biochemical microenvironment of native cartilage. By orchestrating precise physicochemical cues, they address the limitations of conventional cartilage scaffolds, shifting the paradigm from passive substitution to dynamic remodeling guided by ECM mimicry and stem cell fate modulation.

The revolutionary impact of recombinant protein hydrogels in bone engineering manifests in a paradigm shift from “material-driven regenerative medicine” to “intelligently programmed biological manufacturing” [285]. Spanning molecular-level sequence design to macroscale osseous construct fabrication, this field bridges synthetic biology with bone-specific regenerative strategies. Through the evolution from static scaffold implantation to dynamic bone-mimetic microenvironment engineering, these hydrogels enable precise spatiotemporal control over osteogenesis, vascularization, and mineralization [286]. With advancements in multimodal biomanufacturing platforms and AI-assisted design tools, recombinant protein hydrogels are poised to decode complex hierarchical bone regeneration mechanisms, paving the way for transformative breakthroughs in functional skeletal tissue reconstruction [287].

Hydrogels fabricated through non-covalent interactions, enzymatic crosslinking, or protein–polymer conjugation exhibit substantial potential in the aforementioned three domains. Recently, enzyme-instructed self-assembly (EISA) has emerged as a transformative strategy in oncological therapy. EISA leverages the aberrant enzymatic activity within pathological microenvironments to induce supramolecular hydrogelation, generating dynamic nanostructures capable of simultaneously targeting multiple cancer hallmarks. Crucially, the therapeutic efficacy of EISA depends on the intrinsic self-assembly propensity of molecular precursors. For example, Feng et al. systematically evaluated N-terminal-capped D-tetrapeptides containing phosphotyrosine or diester motifs and identified a strong correlation between anticancer activity and assembly capability—regardless of backbone stereochemistry or regiochemistry [288]. This approach exploits overexpressed cellular enzymes to trigger intracellular assembly of small molecules into functional nanostructures, thereby bypassing drug efflux, disrupting DNA repair pathways, and enabling mitochondrial targeting to enhance chemotherapeutic outcomes. Li et al. also exemplified this strategy using alkaline phosphatase (ALP) to dephosphorylate precursors (Nap-GFFpYSV and Nap-GFFpYIGSR), triggering the self-assembly and hydrogelation of hydrophobic bioactive peptides (tyrosylvaline/YSV and laminin pentapeptide/YIGSR) [289].

**Figure 9:**
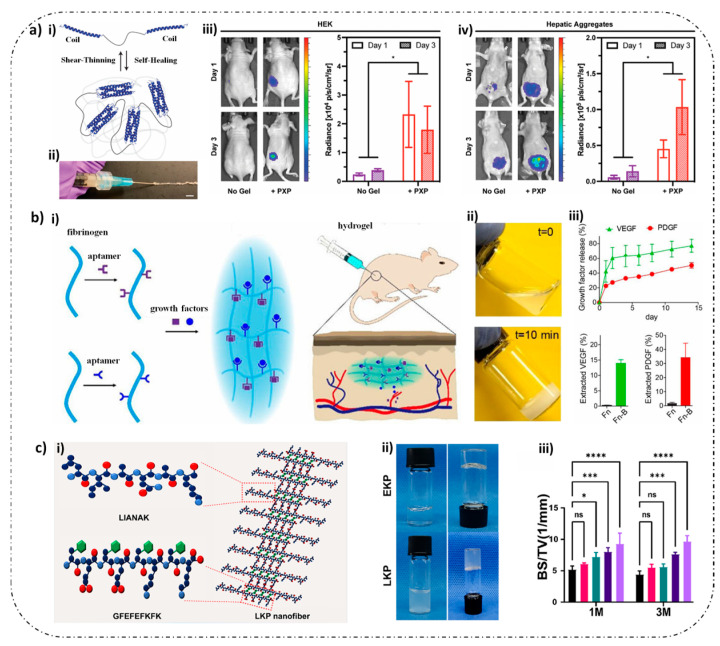
Biomedical applications of recombinant protein-based hydrogels. (**a**) XTEN-based coiled-coil telechelic protein hydrogel. (**i**) Schematic illustration of hydrogel formation via coiled-coil interactions and XTEN domains, exhibiting shear-thinning and self-healing properties. (**ii**) The hydrogel rapidly reassembles after extrusion through high-gauge syringe needles. (**iii**) Quantification of luminescence radiance in HEK293 cells stably expressing luciferase. An unpaired *t*-test was implemented for injection condition comparison (* *p* < 0.05). (**iv**) Parallel analysis in hepatic cell aggregates with luciferase expression driven by a modified albumin promoter. An unpaired *t*-test was implemented for injection condition comparison (* *p* < 0.05). Reproduced with permission from ref. [243]. Copyright 2024, Elsevier. (**b**) Dual-aptamer-functionalized injectable fibrin hydrogel. (**i**) Schematic of aptamer-functionalized fibrin hydrogel formation and its role in promoting angiogenesis. (**ii**) Visual appearance of the hydrogel at different time points post-injection (t = 0 and t = 10 min). (**iii**) Controlled-release profiles of VEGF and PDGF-BB: top panel shows sequential release over 14 days; bottom panel quantifies residual VEGF and PDGF-BB content on Day 14. Reproduced with permission from ref. [251]. Copyright 2019, American Chemical Society. (**c**) SF hydrogel composite scaffold. (**i**) Schematic showing peptide sequence design and β-sheet-driven self-assembly into nanofiber-based SF hydrogels. (**ii**) Macroscopic view of recombinant silk fibroin scaffolds prepared using different peptide sequences. (**iii**) Quantitative analysis of subchondral bone regeneration using micro-CT imaging after 1 and 3 months of implantation. Quantitative analysis of subchondral bone based on micro-CT images (* *p* < 0.05, *** *p* < 0.001, **** *p* < 0.0001, and ns indicates no significant difference). Reproduced with permission from ref. [282]. Copyright 2024, Wiley-VCH GmbH.

Complementing these innovations, recombinant protein hydrogels—applied across applications ranging from smart wound dressings to biomimetic bone scaffolds—are redefining the paradigm from passive structural support to programmable functional systems. By integrating dynamic crosslinking chemistries, spatiotemporally controlled release mechanisms, and multiscale fabrication strategies, these hydrogels effectively address the limitations of traditional platforms (Figure 9). When synergized with advances in synthetic biology and artificial intelligence, recombinant protein hydrogels accelerate the decoding of organ fabrication blueprints, ushering in a new era of programmable living systems for precision regenerative medicine.

## 6. Challenges and Future Trends

As genetically engineered, highly customizable biomaterials, recombinant protein hydrogels face multiple technical and translational bottlenecks in biomedical applications. Their design and production rely on the precise orchestration of amino acid sequences, domain folding, and self-assembly kinetics—complex processes that are influenced by gene construct design, expression system selection, and purification strategies. These intricacies not only lead to high production costs but also contribute to batch-to-batch variability, posing significant challenges for scalable manufacturing. From a material performance perspective, although rational sequence design enables the modulation of mechanical properties, porosity, and degradation kinetics, dynamic crosslinking networks still struggle to maintain mechanical stability under high-load conditions. Moreover, excessive integration of functional domains may disrupt protein folding fidelity and self-assembly efficiency, limiting the material’s overall performance. Biologically, the use of non-humanized expression systems may result in residual endotoxins or the absence of key post-translational modifications, potentially triggering adverse immune responses. In addition, the long-term biosafety of degradation byproducts remains inadequately understood. Clinically, recombinant protein hydrogels encounter barriers such as insufficient vascularization, suboptimal synchronization between degradation rates and tissue regeneration timelines, and unresolved challenges in sterilization, shelf-life stability, and large-scale standardization. As biologics, they also face stringent quality control requirements, and the current lack of long-term, large-animal implantation data further restricts their path to clinical translation and industrialization. Overcoming these challenges will require cross-disciplinary breakthroughs in molecular engineering, bioprocess optimization, and translational evaluation systems [290,291,292,293,294].

Looking ahead, the development of recombinant protein hydrogels will increasingly focus on precision molecular design and multifunctional integration. Artificial intelligence-guided optimization of dynamic response modules and gene-editing strategies to enhance folding efficiency will drive the emergence of intelligent hydrogel networks capable of actively synchronizing with the biological processes of tissue regeneration. Functionally, next-generation systems will incorporate chemokines, anti-inflammatory agents, and gene-editing elements, combined with advanced manufacturing techniques such as microfluidic-assisted 3D printing to recapitulate organ-level structural and functional heterogeneity. Clinically, key priorities will include mechanical reinforcement strategies, injectable and in situ-forming systems, and temporally programmable drug release platforms. These advances must be supported by rigorous long-term in vivo safety assessments, particularly concerning degradation byproducts. Industrial translation will depend on innovations in continuous-flow biomanufacturing, cryopreservation techniques, and sterilization methods, along with the establishment of biologic-specific quality assurance systems. In the broader scope, interdisciplinary integration will extend into emerging fields such as flexible bioelectronics and metabolically active programmable scaffolds. Through sustainable production approaches and improved ethical governance, recombinant protein hydrogels are poised to bridge the gap from tailor-made biomaterials to dynamically responsive, living therapeutic systems—ushering in a new era of intelligent, regenerative medicine.

Recombinant protein hydrogels have progressed from preclinical studies into early-phase human trials. As of 2025, notable candidates include injectable biphasic hydrogels for ankle cartilage repair (NCT0602876), adhesion molecule-loaded hydrogels for periodontal intraosseous defects delivered via minimally invasive surgery (NCT05653245), and antibiotic-eluting hydrogel-coated implants for secondary prevention of chronic periprosthetic hip infections utilizing a comparative single- versus two-stage surgical approach (NCT04251377). Persistent translational hurdles include manufacturing scale-up, Th2-biased immune responses to degradation byproducts in primate models, and regulatory uncertainties surrounding stimuli-responsive materials. Overcoming these challenges through advances in scalable bioprocessing, immunomodulatory design, and clear regulatory pathways will be essential to establish recombinant protein hydrogels as mainstream clinical therapies.

## 7. Conclusions

This review systematically elucidates the core advantages of recombinant protein hydrogels as intelligent biomaterials, in which genetically programmable protein backbones establish the molecular basis for tailoring physical and chemical crosslinking mechanisms to balance dynamic reversibility with mechanical stability. Through modular molecular engineering strategies, these materials acquire spatiotemporally precise functions—including controlled drug release, directed cellular modulation, and an adaptive tissue microenvironment—demonstrating transformative potential in tissue regeneration scaffolds, targeted delivery systems, and biomanufacturing. However, clinical translation remains constrained by challenges such as scalable manufacturing bottlenecks and limited understanding of long-term in vivo metabolic behavior. Future progress will require the integration of computational design with multiscale fabrication technologies to evolve these programmable scaffolds into closed-loop, theranostic platforms.

## Figures and Tables

**Table 1:** Comparative analysis of crosslinking mechanisms.

Category	Mechanism	Principle	Advantages	Disadvantages	Recent Examples	References
Physical Crosslinking	One-component coiled-coil self-assembly	Oligomerization via hydrophobic residues in α-helices	Tunable stiffness, viscoelasticity, degradation	Prone to intramolecular cyclization; low strength	Leucine zipper substitutions coupled at both termini of the GB1 protein to engineer an A(G)_8_A motif	[85,86,87,88,89]
	Stimulus-responsive	Conformational changes triggered by temperature/pH/magnetic field	Spatiotemporal control; injectability	Complex synthesis; potential cytotoxicity	Physical blending of PF and SF; collagen–PEG–cysteine conjugate	[33,90,91,92,93,94]
	Protein–protein/peptide interaction	Biomolecular recognition	High specificity; dynamic self-assembly	Limited mechanical strength	TPR-DESVD protease-responsive cleavage system; complementary WW domain/proline-rich peptide recognition motif	[100,101,102]
Chemical Crosslinking	Tyrosine-mediated crosslinking	Oxidative coupling forming dityrosine bonds	Biocompatible; enzyme/light-triggered	Slow gelation; requires aerobic environment	Recombinant spider silk protein; tyrosine-rich resilin-mimetic protein	[106,107]
	Lysine-mediated crosslinking	Schiff base/Michael addition reactions	Rapid gelation; tunable crosslink density	Crosslinker toxicity	Site-specific conjugation of EGCG to recombinant SF; lysine-rich recombinant ELP	[108,109]
	Cysteine-mediated crosslinking	Disulfide bonds/thiol reactions	Dynamic reversibility; photoresponsiveness	Sensitive to oxidative environments	Recombinant SF-ELP fusion protein; Cys-P4-Cys β-sheet forming protein; S-Ag coordination in recombinant ELPs	[111,112,113]
	Enzyme-mediated crosslinking	LOX/HRP catalytic oxidation crosslinking	Biospecific; mild conditions	High enzyme cost; batch variability	Lysine/hydroxylysine residues in collagen and elastin; tyrosine residues in SF	[115,117,119]
	Isopeptide bond ligation	Covalent conjugation via SpyTag/SpyCatcher or split intein	Bioorthogonal; modular design	Genetic engineering complexity	SpyCatcher/SpyTag-mediated fusion of fluorescent tags, RGD motifs, and LIF	[121,122,126]
Interpenetrating Networks	Protein–protein/polysaccharide/polymer	Physical interpenetration of multiple networks	Mechanical reinforcement; multifunctionality	Complex fabrication; potential phase separation	Type I collagen/recombinant spider silk eADF4(C16)-RGD composite; fibrin–alginate covalent conjugate; PEGylated collagen conjugate	[10,128,129,130,131,132,135,136]

**Table 3:** Comparative analysis of four molecular engineering modules for hydrogel design.

Category	Mechanism	Principle	Advantages	Disadvantages	Recent Examples	References
Self-Assembly Module	Peptide amphiphiles	Assembly driven by hydrophobic/hydrophilic domain segregation	Spontaneous formation; high bioactivity	Low mechanical strength	Conjugation of A_2_G_2_ peptide to VEGF-mimetic QK peptide; V2A2 peptide coupled to mitochondria-targeting SS-31 peptide	[152,153]
	Surfactant-like peptides	Surfactant-mimetic structure (hydrophilic head/hydrophobic tail)	High biocompatibility; membrane permeability	Low stiffness	Amphiphilic recombinant protein [(C(18))2K]2KR8GRGDS	[154]
Responsive Module	pH-responsive	Charge switching via protonation/deprotonation	Targeted delivery	Narrow operational pH range	Recombinant humic acid-mimetic hydrogel	[181,182]
	Thermoresponsive	LCST/UCST phase transition	Injectable; controllable gelation	Hysteresis effects	PEG/ELP hybrid hydrogel	[178,185]
	Photoresponsive	Photo-triggered crosslinking	Spatiotemporally precise control	Limited tissue penetration depth	Recombinant SF/collagen composite hydrogel	[186,187]
	Ultrasound-responsive	Ion release/enzyme activation via cavitation	Deep tissue penetration	External equipment dependency	Fibrinogen-based hydrogel	[191,192]
Chemical Modification Module	Site-specific modification	Targeted chemical conjugation to specific residues (e.g., Tyr, Lys)	Precise functional group introduction	Potential protein folding interference	Collagen hydrogel modified with MA and SA; tyrosine-selective modification of ELPs with PTAD	[202,204]
	Photosensitive group incorporation	Photocleavable groups/photosensitizer conjugation	Light-controlled drug release/degradation	Potential phototoxicity	ELP scaffold with PS-conjugated hydrogels via methionine-specific oxidation	[205]
	Cleavable linkers	Hydrolysis-sensitive bond design	Controlled protein drug release	Synthetic complexity	Lysozyme–PEG hybrid hydrogel	[206]
Functionalization Module	Adhesive motifs	Fusion of bioactive peptides (e.g., RGD)	Enhanced cell proliferation/angiogenesis	High cost; stability issues	Fibronectin-derived ligand (RGD/HB) and EGF-conjugated hydrogel	[207,208]
	Dynamic remodeling	Integration of matrix metalloproteinase-2 (MMP-2)	Spatiotemporally controlled remodeling	Long-term safety undetermined	MMP-2-cleavable PEG hydrogel	[209,210]
	Antimicrobial components	Incorporation of antimicrobial peptides/proteins	Broad-spectrum antibiofilm activity	Potential resistance development	Tet213 antimicrobial peptide-grafted GelMA	[211,212,213]
	Immunomodulatory elements	Conjugation of immune factors	Macrophage polarization/anti-inflammatory regulation	Complex in vivo mechanisms	CGRP-functionalized GelMA hydrogel	[214,215]

**Table 4:** Functionalization strategies and key outcomes of recombinant protein hydrogels applied in biomedicine.

Application Field	Clinical Application	Protein Scaffold	Functionalization Strategy	Key Outcomes	Reference
Tissue Engineering	Skin regeneration	SF/sericin composite hydrogel	DMPG crosslinking	Enhanced antibacterial efficacy, accelerated wound closure	[4]
	Wound healing	Collagen-like protein	RC conjugated with GelMA to form GelMA-RC hydrogel	Enhanced cell viability and migration, accelerated wound healing	[222]
	Skin wound regeneration	Recombinant humanized type III collagen	Composite hydrogel composed of SA@MnO_2_ nanoparticles, RHC, and MSCs	Enhanced cell proliferation/migration, accelerated re-epithelialization, improved collagen deposition, promoted neovascularization	[223]
	Scar elimination in skin wounds	Silk fibroin	Recombinant NT3 hydrogel fused with SFL	Accelerated wound healing with induced folliculogenesis	[224]
	Skin aging	Recombinant collagen	THPC-crosslinked recombinant collagen	Enhanced proliferation, adhesion, and migration of HFF-1; increased dermal density, improved skin elasticity, reduced transepidermal water loss	[225]
Drug Delivery Systems	Long-term protein delivery	Recombinant spider silk protein	Encapsulation of ARPE-19 cells	Sustained delivery of therapeutic cellular secretions	[226]
	Drug delivery	Apoferritin	Polylysine-modified apoferritin with four lysine residues	Suppressed chondrocyte apoptosis, enhanced cell migration, improved cartilage protection, alleviated osteoarthritis symptoms	[227]
	TME-responsive delivery	Collagen/hyaluronic acid	pH-sensitive Schiff base bonds	Targeted drug release in acidic environments, significant tumor suppression	[90]
Bone Regeneration Engineering	Osteoarthritis therapy	MMP-2/collagen II	Methacrylation	Inhibited macrophage activation, reduced pro-inflammatory cytokines, protected chondrocytes	[228]
	Personalized bone defects	Collagen	rhBMP-2 loading	Significant osteogenic effects, enhanced osseointegration	[229]
	Bone regeneration acceleration	Recombinant human collagen	rhCol/bFGF hydrogel prepared via TG crosslinking	Achieved sustained bFGF release, accelerated bone regeneration	[230]

## Data Availability

No new data were created or analyzed in this study.

## Abbreviations

The following abbreviations are used in this manuscript:

ECM	extracellular matrix
PAAm	polyacrylamide
PEG	polyethylene glycol
3D	three-dimensional
ELPs	elastin-like polypeptides
SF	silk fibroin
RLPs	resilin-like polypeptides
MFPs	mussel foot proteins
IDP	intrinsically disordered protein
LOX	lysyl oxidase
LCST	lower critical solution temperature
Tt	transition temperature
UCST	upper critical solution temperature
PBS	phosphate-buffered saline
DOPA	3,4-dihydroxy-L-phenylalanine
CLPs	collagen-like peptides
ASCs	adipose-derived stem cells
PLA	polylactic acid
PF	Pluronic F-127
Col	collagen
MNPs	magnetic nanoparticles
RTX	repeat-in-toxin
TPR	tetratricopeptide repeat
HRP	horseradish peroxidase
H2O2	hydrogen peroxide
ROS	reactive oxygen species
Ru	ruthenium
SPS	sodium persulfate
EGCG	(-)-epigallocatechin-3-O-gallate
GA	glutaraldehyde
OHA	oxidized hyaluronic acid
PAs	peptide amphiphiles
SELPs	silk fibroin–ELPs
S-Ag	sulfur–silver
Hyl	hydroxylysine
CaO2	calcium peroxide
SA	silk acid
LIF	leukemia inhibitory factor
INs	interpenetrating networks
mTG	microbial transglutaminase
HA	hyaluronic acid
VEGF	vascular endothelial growth factor
pG	protein G
SLPs	surfactant-like peptides
polyD	polyaspartic acid
bRGD	bone salivary protein-derived RGD peptide
HUVECs	human umbilical vein endothelial cells
PTAD	phenyltriazolinedione
SPAAC	strain-promoted alkyne–azide cycloaddition
MA	methacrylic anhydride
SA	succinic anhydride
BMSCs	bone marrow mesenchymal stem cells
sGAG	glycosaminoglycan
PS	photosensitizer
1O2	singlet oxygen
FAK	focal adhesion kinase
EGF	epidermal growth factor
MMP-2	metalloproteinase-2
AMPs	antimicrobial peptides
GelMA	gelatin methacryloyl
hDPSCs	human dental pulp stem cells
PPARγ	peroxisome proliferator-activated receptor gamma
PTT	photothermal therapy
PDT	photodynamic therapy
EPL	ε-poly-L-lysine
LPS	lipopolysaccharides
CGRP	calcitonin gene-related peptide
DMPG	1,2-dimyristoyl-sn-glycero-3-phosphoglycerol
EVs	extracellular vesicles
rhCol III	recombinant human type III collagen
MSC	mesenchymal stem cell
PAD	peripheral artery disease
PGRN	progranulin
DIC	diclofenac
SFH	silk fibroin hydrogels
rhBMP-2	recombinant human bone morphogenetic protein-2
CAD/CAM	computer-aided design/manufacturing
ZrO2	zirconia
β-TCP	β-tricalcium phosphate
THRC	triple-helical recombinant collagen
BMP-2	bone morphogenetic protein-2
OCMC	oxidized carboxymethyl cellulose
NSC	N-succinyl chitosan
hTGF-β1	human transforming growth factor-β1
hBM-MSCs	human bone marrow mesenchymal stem cells
SF-GMA	glycidyl methacrylate-modified silk fibroin
CAR-T	Chimeric Antigen Receptor T-Cell Immunotherapy
RC	collagen-like protein
RHC	recombinant humanized type III collagen
MSCs	mesenchymal stem cells
NT3	neurotrophin-3
SFL	SF light chain
Col3	type III collagen
THPC	tetrakis(hydroxymethyl)phosphonium chloride
HFF-1	human foreskin fibroblasts-1
ENG	ELP nanogel
CXB	celecoxib
TG	transglutaminase
EISA	enzyme-directed self-assembly
ALP	alkaline phosphatase
